# Supplementary material for: Trichoplax adhaerens reveals a network of nuclear receptors sensitive to 9-cis-retinoic acid at the base of metazoan evolution
Source: PeerJ. 2017 Sep 29;5:e3789. doi: 10.7717/peerj.3789 (PMC5624297; doi:10.7717/peerj.3789)
Supplement: File S1 [file peerj-05-3789-s001.docx]

**Supplementary file S1. Sequences for ClustalO alignment of RXR sequences from selected species shown in Fig.1.**

(in FASTA format)

>sp|P20153|USP_DROME Protein ultraspiracle OS=Drosophila melanogaster GN=usp PE=1 SV=1

------------------------------------------------------------

--------------MDNCDQDASFRLSHIKEEVK-------PDISQLNDSNNSS------

---------------FSPKAESPVPFMQAMSMVHVL-----------PGSNSA-------

SSNNNSAGDAQ-MAQAPNSAGGSAAAAVQQQYPPNHPLSGSKHLCSICGDRASGKHYGVY

SCEGCKGFFKRTVRKDLTYACRENRNCIIDKRQRNRCQYCRYQKCLTCGMKREAVQEERQ

RGARNAAGRLSASGGGSSGPGSVGGSSSQGGGGGGGVSGGMGSGNGSDDFMTNSVSRDFS

IERIIEAEQRAETQCGDRALTFLRV-GPYSTVQPDYKGAVSALCQVVNKQLFQMVEYARM

MPHFAQVPLDDQVILLKAAWIELLIANVAWCSIVSLDDGGAGGGGGGLGHDGSFERRSPG

LQPQQLFLNQSFSYHRNSAIKAGVSAIFDRILSELSVKMKRLNLDRRELSCLKAIILYNP

DIRGIKSRAEIEMCREKVYACLDEHCRLEHPGDDGRFAQLLLRLPALRSISLKCQDHLFL

FRITSDRPLEELFLEQLEAPPPPGLAMKLE

>tr|S5ZWR0|S5ZWR0_AURAU Retinoid X receptor OS=Aurelia aurita GN=RXR PE=2 SV=1

---------------MEISYCDST------------------------------------

-----------------TDR----------EELH--LP---EKVPGME------Y----A

CEE----------------------DGSSMNMVDSC-------LPEPPPLESI----DSY

SPLSGSDGTPGSSSS-------SLSFFPQNSSPNSNGSERQMLPCAVCSDKAYVKHYGVV

ACEGCKGFFKRSVRNNRKYQCLGNQRCDIDRKSRNKCQYCRFQKCIEVGMKPEAVQDETL

KKEKRESTKRKAANPVSSGSK---GSP-------------------VEVTSSRVEMPLVP

IDLVVSAESMVEPSIQ----LFA----------NTAVDPIRHVCLAADKQLASLAEWAKK

LPHFTSLDITDQVVLLQWSWPELLIGGFCHRSAAVR------------------------

---DGILLATGLHLTRENLKKAGVGAIIDKIFAEVIEKMQEMQLDRAEWGCLRAVMLFSP

DAKNLKDVQQVETYREMYSATLEDYMKKNRPDQPDRFTKVILRVPALKSIGLQALEHLYF

FKLIGDVPMETFLLDMLEVTQP--------

>tr|O96562|O96562_TRICY Retinoic acid X receptor OS=Tripedalia cystophora PE=2 SV=1

---------------MA-VQCNSS------------------------------------

-----------------TANDVVSKEVSEETKLQ--IV---KEEETSAPSCDSSV----S

AMS----------------------KEGGLAMVDSC-------LKEASPLESI----HPY

SPLASDASGSSTSPI-------ASSSLLQ-LPSLTADSQRPVQPCSVCSDKAYVKHYGVF

ACEGCKGFFKRSVRNNRKYSCLGKRHCDTDKKSRNRCQYCRFQKCVQVGMKPEAVQDETL

KKERKDYRKRL-----PSTPK---GSP-------------------AEVTSSKVDLPMIP

IESIIAAETLVDPGIQ----TFA----------SANTDPIRHVCLAADKQLASLAEWAKR

LPHFRDLSIADQVVLLQWSWPELLIGGFCHRSCAVK------------------------

---DGILLSTGLHLTRDNLKKAGVGAIIDKIFSEVIEKMQEIQMDRAEWGCLRAIMLFSP

DAKGLTAIDQVENYRELYTSTLEDHVKRKHPEQPDRFTKVILRIPALKSIGLQALEHLYF

FKLIGDVPMDTFLLDMLEVDRS--------

>TaRXR ID 53515

------------------------------------------------------------

------------------------------------------------------------

------------------------------------------------------------

--------------------------MEDRSFKKEAQADKSNGNCLVCNDRASGRHYGVY

SCEGCKGFFKRTVRKNLTYTCRDNRNCDIDKKQRNRCQYCRYQKCLQVGMKQEAVQEERV

KNSTPTSKTTLPIAIAD-------G-----------------------FPLPTYGNDEMP

VEAIRDAESTLNMNSV----PYVEM----------QSNPVLNICQAADKQLFNLVEWAKK

IPHFCDLCVDDQVILLRSGWNELLIAAFSFRSIAVE------------------------

---DGLLLSTGHYIHRTSAHNAGIGAIFDRILTELVNQMRYLKMDKTELGCLRAIILFNP

DVRGLTSADRVEKYRELVYGALEAYVKKRFPDQLCRFAKLLLRLPALRAISLKTLEHLFF

YKLIGDPPIDTFLMEMLETGSS*-------

>sp|P28702|RXRB_HUMAN Retinoic acid receptor RXR-beta OS=Homo sapiens GN=RXRB PE=1 SV=2

MSWA--ARPPFLPQRHAAGQCGPVGVRKEMHCGVASRWRRRRPWLDPAAAAAAAVAGGEQ

QTPEPEPGEAGRDGMGDSGRDSRSPDSSSPNPLPQGVPPPSPPGPPLPPSTAPSLG----

----GSGAPP-------PPPMPPPPLGSPFPVISSSMGSPGLPPPAPPGFSGPVSSPQIN

STVSLPGGGSGPPEDVKPPVLGVRGL---HCPPPPGGPGAGKRLCAICGDRSSGKHYGVY

SCEGCKGFFKRTIRKDLTYSCRDNKDCTVDKRQRNRCQYCRYQKCLATGMKREAVQEERQ

RGKDKDG-D---------------------------------------GEGAGGAPEEMP

VDRILEAELAVEQKSD----QGVEGPGGTGGSGSSPNDPVTNICQAADKQLFTLVEWAKR

IPHFSSLPLDDQVILLRAGWNELLIASFSHRSIDVR------------------------

---DGILLATGLHVHRNSAHSAGVGAIFDRVLTELVSKMRDMRMDKTELGCLRAIILFNP

DAKGLSNPSEVEVLREKVYASLETYCKQKYPEQQGRFAKLLLRLPALRSIGLKCLEHLFF

FKLIGDTPIDTFLMEMLEAPHQLA------

>sp|P48443|RXRG_HUMAN Retinoic acid receptor RXR-gamma OS=Homo sapiens GN=RXRG PE=1 SV=1

---------------------------------MYGNYSHFMKFPAGY--------GGS-

-------------------------------PGHTGS-------TSMSPSAALSTGKPMD

SHPSYTDTPVSAPRTLSAVGTPLNALGSPYRVITSAMGPPSGALAAPPGINLVAPPSSQL

NVVNS----VSSSEDIKPL-PGLPGIGNMN-YPSTSPGSLVKHICAICGDRSSGKHYGVY

SCEGCKGFFKRTIRKDLIYTCRDNKDCLIDKRQRNRCQYCRYQKCLVMGMKREAVQEERQ

RSRERAESE---------------------------------------AECATSGHEDMP

VERILEAELAVEPKTE----SYGDM--NME---NSTNDPVTNICHAADKQLFTLVEWAKR

IPHFSDLTLEDQVILLRAGWNELLIASFSHRSVSVQ------------------------

---DGILLATGLHVHRSSAHSAGVGSIFDRVLTELVSKMKDMQMDKSELGCLRAIVLFNP

DAKGLSNPSEVETLREKVYATLEAYTKQKYPEQPGRFAKLLLRLPALRSIGLKCLEHLFF

FKLIGDTPIDTFLMEMLETPLQIT------

>sp|P19793|RXRA_HUMAN Retinoic acid receptor RXR-alpha OS=Homo sapiens GN=RXRA PE=1 SV=1

-----MDTKHFLP----------L--------------------------------DFST

QVNS----------------------S-LTSPTGRGS----MAAPSLHPSLGP-------

----GIGSPGQLHSPISTLSSPINGMGPPFSVISSPMGPHSMSVPTTPTLGFSTGSPQLS

SPMNP----VSSSEDIKPP-LGLNGVLKVPAHPSGNMASFTKHICAICGDRSSGKHYGVY

SCEGCKGFFKRTVRKDLTYTCRDNKDCLIDKRQRNRCQYCRYQKCLAMGMKREAVQEERQ

RGKDRNENE---------------------------------------VESTSSANEDMP

VERILEAELAVEPKTE----TYVEA--NMGLNPSSPNDPVTNICQAADKQLFTLVEWAKR

IPHFSELPLDDQVILLRAGWNELLIASFSHRSIAVK------------------------

---DGILLATGLHVHRNSAHSAGVGAIFDRVLTELVSKMRDMQMDKTELGCLRAIVLFNP

DSKGLSNPAEVEALREKVYASLEAYCKHKYPEQPGRFAKLLLRLPALRSIGLKCLEHLFF

FKLIGDTPIDTFLMEMLEAPHQMT------

>sp|P51128|RXRA_XENLA Retinoic acid receptor RXR-alpha OS=Xenopus laevis GN=rxra PE=1 SV=1

MSSAAMDTKHFLP----------LGGRT-----CADTLRCTTSWTAGY--------DFSS

QVNS----------------------SSLSSSGLRGS----MTAPLLHPSLGN------S

GLNNSLGSPTQLPSP---LSSPINGMGPPFSVISPPLGP-SMAIPSTPGLGYGTGSPQIH

SPMNS----VSSTEDIKPP-PGINGILKVPMHPSGAMASFTKHICAICGDRSSGKHYGVY

SCEGCKGFFKRTVRKDLTYTCRDSKDCMIDKRQRNRCQYCRYQKCLAMGMKREAVQEERQ

RGKERNENE---------------------------------------VESSNSANEDMP

VEKILEAEHAVEPKTE----TYTEA--NMGLAPNSPSDPVTNICQAADKQLFTLVEWAKR

IPHFSELPLDDQVILLRAGWNELLIASFSHRSIAVK------------------------

---DGILLATGLHVHRNSAHSAGVGAIFDRVLTELVSKMRDMQMDKTELGCLRAIVLFNP

DSKGLSNPLEVEALREKVYASLEAYCKQKYPEQPGRFAKLLLRLPALRSIGLKCLEHLFF

FKLIGDTPIDTFLMEMLEAPHQMT------
