# Supplementary material for: Trichoplax adhaerens reveals a network of nuclear receptors sensitive to 9-cis-retinoic acid at the base of metazoan evolution
Source: PeerJ. 2017 Sep 29;5:e3789. doi: 10.7717/peerj.3789 (PMC5624297; doi:10.7717/peerj.3789)
Supplement: File S2 — ClustalO alignments of HNF4, COUP-TF and ERR sequences from selected species. [file peerj-05-3789-s002.zip › COUP/COUP sequences.docx]

>TaCOUP

MSTRSSEYIDSPTAAAKDETKSLSKELCCLICGDRSNGRHYGVISCEGCKGFFKRSVRRNMKYACTCSANACKITKANRNQCQFCRLQKCFKVGMRKEAVQKERHTSTIRADRNSGKTEKEMTPDSETAINSLIKNLVAAETLVLSSRSLQLQSGFIGFEAICQSSMRILYSVVEWTVKLPYFSEMTSCTDQMTLLRSCWSELFILNAAQWSPPLNMFPYSTTSNFYLTHPQEVMHHICLFQEAIVKLKKRFIDTTEFSCLKALILFNPDVRGLVNPNYVEYIQENIQCALKQHVKSQYPDQPSRFGYLLLRLLMLRSISSKVIEEIFFTSVLCRRSIDIFLCEAMESVKRA*

>sp|P24468|COT2_HUMAN COUP transcription factor 2 OS=Homo sapiens GN=NR2F2 PE=1 SV=1

MAMVVSTWRDPQDEVPGSQGSQASQAPPVPGPPPGAPHTPQTPGQGGPASTPAQTAAGGQ

GGPGGPGSDKQQQQQHIECVVCGDKSSGKHYGQFTCEGCKSFFKRSVRRNLSYTCRANRN

CPIDQHHRNQCQYCRLKKCLKVGMRREAVQRGRMPPTQPTHGQFALTNGDPLNCHSYLSG

YISLLLRAEPYPTSRFGSQCMQPNNIMGIENICELAARMLFSAVEWARNIPFFPDLQITD

QVALLRLTWSELFVLNAAQCSMPLHVAPLLAAAGLHASPMSADRVVAFMDHIRIFQEQVE

KLKALHVDSAEYSCLKAIVLFTSDACGLSDVAHVESLQEKSQCALEEYVRSQYPNQPTRF

GKLLLRLPSLRTVSSSVIEQLFFVRLVGKTPIETLIRDMLLSGSSFNWPYMAIQ

>sp|P10589|COT1_HUMAN COUP transcription factor 1 OS=Homo sapiens GN=NR2F1 PE=1 SV=1

MAMVVSSWRDPQDDVAGGNPGGPNPAAQAARGGGGGAGEQQQQAGSGAPHTPQTPGQPGA

PATPGTAGDKGQGPPGSGQSQQHIECVVCGDKSSGKHYGQFTCEGCKSFFKRSVRRNLTY

TCRANRNCPIDQHHRNQCQYCRLKKCLKVGMRREAVQRGRMPPTQPNPGQYALTNGDPLN

GHCYLSGYISLLLRAEPYPTSRYGSQCMQPNNIMGIENICELAARLLFSAVEWARNIPFF

PDLQITDQVSLLRLTWSELFVLNAAQCSMPLHVAPLLAAAGLHASPMSADRVVAFMDHIR

IFQEQVEKLKALHVDSAEYSCLKAIVLFTSDACGLSDAAHIESLQEKSQCALEEYVRSQY

PNQPSRFGKLLLRLPSLRTVSSSVIEQLFFVRLVGKTPIETLIRDMLLSGSSFNWPYMSI

QCS

>tr|Q91720|Q91720_XENLA Coup transcription factor OS=Xenopus laevis GN=nr2f5 PE=2 SV=1

MAMVVNPWQEDIPGVPGSQMNNPPGLCNQDPGGTPQTPTTPKGGIPGQDPVHSGDKGVPN

VDCLVCGDKSSGKHYGQFTCEGCKSFFKRSVRRNLTYTCRSNRDCPIDQHHRNQCQYCRL

KKCLKVGMRREVQRGRMSHPQTSPGQYTLNNVDPYNGHSYLTGFISLLLRAEPYPTSRYG

AQCLQPNNIMGIENICELAARLLFSAIEWAKNIPFFPDFQLSDQVSLLRMTWSELFVLNA

AQCSMPLHVAPLLARAGLHASPMSADRVVAFMDHIRIFQEQVEKLKALHVDSAEYSCLKA

IALFTPDAVGLSDIGHVESIQEKSQCALEEYVRNQYPNQPTRFGRLLLRLPSLRIVSAPV

IEQLFFVRLVGKTPIETLIRDMLLSGSSFNWPYMPMQ

>sp|P16376|7UP2_DROME Steroid receptor seven-up, isoform A OS=Drosophila melanogaster GN=svp PE=2 SV=3

MCASPSTAPGFFNPRPQSGAELSAFDIGLSRSMGLGVPPHSAWHEPPASLGGHLHAASAG

PGTTTGSVATGGGGTTPSSVASQQSAVIKQDLSCPSLNQAGSGHHPGIKEDLSSSLPSAN

GGSAGGHHSGSGSGSGSGVNPGHGSDMLPLIKGHGQDMLTSIKGQPTGCGSTTPSSQANS

SHSQSSNSGSQIDSKQNIECVVCGDKSSGKHYGQFTCEGCKSFFKRSVRRNLTYSCRGSR

NCPIDQHHRNQCQYCRLKKCLKMGMRREAVQRGRVPPTQPGLAGMHGQYQIANGDPMGIA

GFNGHSYLSSYISLLLRAEPYPTSRYGQCMQPNNIMGIDNICELAARLLFSAVEWAKNIP

FFPELQVTDQVALLRLVWSELFVLNASQCSMPLHVAPLLAAAGLHASPMAADRVVAFMDH

IRIFQEQVEKLKALHVDSAEYSCLKAIVLFTTGKLLDILYKDVPALLTKVSALLGKGSTA

SNDDVLAVVRDHLDELNRQEQESQAQQQAPLHLAAFMNCVAGVEAAVQQAEQAQVPTSSA

SASVSAPLVPSAGSAFSSCQAKSAGSEMDLLASLYAQAQATPPSSGGGDASGHNNSSGLG

ASLPTQSQSGSSSRNLTASPLSTSLATAPAPASASAPAPVPTSSVAQVPVPAPVPVTSSA

SSSSLGGGAYQTPSAAAAAAAMFHYQTPPRAAFGSAFDMFHHSTPFGVGVGHAHALAHSS

GSGSASFGSPSYRYSPYSLAGSRWQL
