# Supplementary material for: Trichoplax adhaerens reveals a network of nuclear receptors sensitive to 9-cis-retinoic acid at the base of metazoan evolution
Source: PeerJ. 2017 Sep 29;5:e3789. doi: 10.7717/peerj.3789 (PMC5624297; doi:10.7717/peerj.3789)
Supplement: File S2 — ClustalO alignments of HNF4, COUP-TF and ERR sequences from selected species. [file peerj-05-3789-s002.zip › ERR/23.09/1-s2.0-S0006291X08015970-main.pdf]

Biochemical and Biophysical Research Communications 375 (2008) 623–627

Contents lists available at ScienceDirect

Biochemical and Biophysical Research Communications

journal homepage: www.elsevier.com/locate/ybbrc

Trichoplax, the simplest known animal, contains an estrogen-related receptor but no estrogen receptor: Implications for estrogen receptor evolution

Michael E. Baker *

Department of Medicine, University of California, San Diego, 9500 Gilman Drive, La Jolla, CA 92093-0693, USA

ARTICLE INFO

Article history:
Received 12 August 2008
Available online 21 August 2008

Keywords:
Estrogen receptor evolution
Invertebrate estrogen receptors
Estrogen-related receptor
Trichoplax
Lophotrochozoan estrogen receptors
Nuclear receptors

ABSTRACT

Although, as their names imply, estrogen receptors [ERs] and estrogen-related receptors [ERRs] are related transcription factors, their evolutionary relationships to each other are not fully understood. To elucidate the origins and evolution of ERs and ERRs, we searched for their orthologs in the recently sequenced genome of *Trichoplax*, the simplest known animal, and in the genomes of three lophotrochozoans: *Capitella*, an annelid worm, *Helobdella robusta,* a leech, and *Lottia gigantea,* a snail. BLAST searches found an ERR in *Trichoplax*, but no ER. BLAST searches also found ERRs in all three lophotrochozoans and invertebrate-like ERs in *Capitella* and *Lottia*, but not in *Helobdella*. Unexpectedly we find that the *Capitella* ER sequence is closest to ERβ, unlike the other invertebrate ER sequences, which are closest to ERα. Our database searches and phylogenetic analysis indicate that invertebrate ERs evolved in a lophotrochozoan and steroid-binding ERs evolved in a deuterostome.

© 2008 Elsevier Inc. All rights reserved.

From the beginning, when estrogen-related receptor α [ERRα] and ERRβ were first cloned [1], the ERR has been an enigma [2,3]. As its name implies, ERR sequences are similar to that of vertebrate estrogen receptors [ER]. The ligand-binding domain of human ER and ERRα and ERRβ have about 35% sequence identity and 60% positive matches, when conservative replacements such as arginine/lysine and glutamic acid/aspartic acid are considered. Yet, these ERRs do not bind estradiol or other steroids [1–3]. Subsequently, ERRγ [4] was cloned and also found to lack steroid-binding activity. Indeed, *a bona fide* biological ligand for an ERR has not yet been identified. As a result, the ERR belongs to the orphan receptor group [5,6] in the nuclear receptor family of transcription factors [7–10].

An explanation for the absence of steroid binding by ERRs came from analysis of the crystal structures of human ERRα [11,12] and ERRγ [13], which showed that the ligand-binding site is too small to accommodate a steroid [11–14]. The crystal structures also showed that in ERRα and ERRγ the activation function 2 (AF2) domain on α-helix 12 is in a conformation for productive interactions with coactivators [15,16], which explains why the ERR does not require a ligand to become transcriptionally active in cell assays [1–5]. In the last few years, there has been progress in beginning to elucidate ERR functions, which include regulating bone formation [2,3,17,18] and mitochondrial biogenesis [18–20].

Complicating understanding of the evolution of ERRs and vertebrate ERs was the cloning in the last five years of several invertebrate ERs from mollusks [21–25]. Invertebrate ERs, such as octopus ER, have about 34% sequence identity and 58% positive matches with the estrogen-binding domain in human ERα, and 29% identity and 56% positive matches with human ERRγ. Similar to ERRs, invertebrate ERs do not bind estradiol with high affinity, in contrast to vertebrate ERs, which are activated by 0.2 nM estradiol [26]. Also, similar to ERRs, invertebrate ERs are constitutively active transcription factors in cell assays [21–25]. A biological function for invertebrate ERs has not been reported.

The phylogenetic relationships of vertebrate and invertebrate ERs to each other and to ERRs are still not fully understood [9,17,18,21,25]. When did the ancestral ER/ERR arise? Was this ancestor more like an ERR or an ER? How did the estrogen-binding vertebrate ER and the constitutively active invertebrate ERs evolve [8,27–29]? That is, did vertebrate and invertebrate ERs evolve from a gene duplication of an ancestral ER, or did the vertebrate and invertebrate ERs evolve from separate ancestral genes? An opportunity to address these questions comes from recent sequencing by the Joint Genome Initiative [http://genome.jgi-psf.org] of genomes of *Trichoplax*, which is considered to be the simplest metazoan [30–32], and of three lophotrochozoans: *Capitella*, a segmented worm, *Helobdella*, a leech and *Lottia*, a snail.

As reported here, BLAST [33] searches found an ERR, but no ER in *Trichoplax*, indicating that ERRs are more ancient than ERs. BLAST searches of the three recently sequenced lophotrochozoan genomes found ERRs in *Capitella*, *Helobdella*, and *Lottia*, and invertebrate ERs in *Capitella* and *Lottia*. The current genome release of *Helobdella* does not contain an invertebrate ER. To our surprise, a BLAST search of GenBank with the *Capitella* ER sequence indicates

* Corresponding author. Fax: +1 858 822 0873.
E-mail address: mbaker@ucsd.edu

0006-291X/$ - see front matter © 2008 Elsevier Inc. All rights reserved.
doi:10.1016/j.bbrc.2008.08.047

that it is closest to ERβ, in contrast to the other invertebrate ER sequences, which are closest to ERα.

The evidence that invertebrate-like ERs are restricted to lophotrochozoans and our phylogenetic analysis of protostome and deuterostome ERRs and ERs indicates that invertebrate ERs share a common ancestor with protostome ERRs, and steroid-binding vertebrate ERs evolved from an ancestor in a deuterostome [28].

Materials and methods

BLAST [33] was used to collect ERR and ER sequences from the JGI server [http://genome.jgi-psf.org] and GenBank. Two different methods, Clustal X 2.0 [34], which uses a neighbor-joining algorithm [35], and PHYML [36], which uses a maximum likelihood algorithm, were used to construct phylogenetic trees of various ERs, ERRs, human retinoid X receptor-α (RXRα) and amphioxus RXR.

For the Clustal X 2.0 phylogeny, the multiple alignments of ERs, ERRs and RXRs were done using the iteration option for each alignment step in the multiple alignment. This alignment was converted to a phylogenetic tree using the neighbor-joining algorithm [35] with a correction of branch lengths for rate heterogeneity between sites.

For PhyML, the Muscle algorithm [37] was used to construct a multiple alignment. PhyML was used with the WAG substitution model [38] and a gamma distribution of rates between sites (four categories, parameter α estimated by PhyML), and 100 bootstrap replicates. The phylogenetic trees with Clustal X 2.0 and PhyML gave similar topologies and bootstrap values.

Accession numbers are human ERRγ [GenBank Accession No. AAQ93376], human ERα [GenBank Accession No. :NP_000116], *Aplysia californica* (California sea hare) ER [GenBank Accession No. AAQ95045], *Capitella* (polychaete worm) ER [jgi|Capca1|170275], *Crassostrea gigas* (Pacific oyster) ER [GenBank Accession No. BAF45381], *Lottia gigantea* (Owl limpet) ER [jgi|Lotgi1|132166], *Marisa cornuarietis* (giant rams horn) ER [GenBank Accession No. ABI97119], *Nucella lapillus* (Atlantic dogwhelk) ER [GenBank Accession No. ABQ96884], *Octopus vulgaris* (Octopus) ER [GenBank Accession No. ABG00286], *Thais clavigera* (rock shell) ER [GenBank Accession No. BAC66480], Human ERβ [GenBank Accession No. 6166154], Xenopus tropicalis ERα [GenBank Accession No. NP_988866] and ERβ [GenBank Accession No. NP_001035101], *Drosophila melanogaster* ERR [GenBank Accession No. NP_729340], *Apis mellifera* ERR [GenBank Accession No. 110756963], Daphnia ERR [jgi|Dappu1|46682], *Capitella* ERR [jgi|Capca1|108381], *Lottia* ERR [jgi|Lotgi1|168715], *Helobdella* ERR [jgi|Helro1|106750], human RXRα [GenBank Accession No. NP_002948] and amphioxus RXR [GenBank Accession No. AAM46151].

Results and discussion

Four nuclear receptors are present in a basal diploblast

The DNA and ligand-binding domains of human ERRγ, human ERα, octopus ER, *Aplysia* ER, *Thais* ER and oyster ER were used as queries for BLAST searches for orthologs in *Trichoplax*, *Capitella*, *Helobdella*, and *Lottia* on the JGI server. The BLAST search of *Trichoplex* with human ERRγ yielded four high scoring nuclear receptors. Searches with human ERα and invertebrate ERs found the same genes in *Trichoplax*. To classify the four *Trichoplax* genes, we used their sequences as queries for BLAST searches of GenBank. This identified *Trichoplex* jgi|Triad1|16711|gw1.23.179.1 as an ortholog of ERR; the other *Trichoplax* genes appear to be orthologs of COUP, RXR, or HNF4 [Table 1]. Thus, genes with similarity to vertebrate ERR, COUP, RXR, and HNF4 are found in a primitive multicellular animal belonging to the phylum Placozoa [30–32].

Our analysis does not exclude the possibility of other nuclear receptors in *Trichoplax* because our BLAST search focused on finding ancestors of ER and ERR. Also nuclear receptor genes may have been lost in *Trichoplax* during its evolution from an ancestral metazoan. Analyses of other simple metazoan genomes will provide a more definitive inventory of nuclear receptors in basal metazoans.

We focused the rest of our analyses on the relationship of the *Trichoplex* ERR-like gene to invertebrate and vertebrate ERRs and ERs. BLAST searches of the JGI server retrieved ERRs from *Capitella*, Helobdella, and *Lottia*, and an invertebrate ER from *Capitella* and *Lottia*. BLAST did not find an invertebrate ER in *Helobdella*.

A BLAST search of the JGI server retrieved an ERR from *Daphnia*, a water flea. BLAST did not find an invertebrate ER in *Daphnia*. We also used BLAST to retrieve invertebrate ER sequences from *A. californica, O. vulgaris, C. gigas,* and two snails: *T.* clavigera [23] and *M. cornuarietis* [22] and ERR sequences from *M. cornuarietis, A. mellifera,* and *D. melanogaster.*

Divergence of Capitella ER from other invertebrate ERs

To better understand the relationship of invertebrate ERs to steroid-binding ERs, we used BLAST to search GenBank with the domain on each invertebrate ER which corresponds to the steroid-

Table 1
Nuclear receptor genes in *Trichoplax*

Gene ID in JGI Databank	Homolog in GenBank	BLAST score	% Identity and % positives, and gaps
jgi|Triad1|16711|gw1.23.179.1<br>ligand-binding domain	Human ERRγ<br>pdb|1KV6|A	6e−55	Identities: 99/222 (44%)<br>positives: 155/222 (69%)<br>gaps: 0/222 (0%)
>jgi|Triad1|49897|fgeneshTA2_pm.<br>C_scaffold_2000050	Human RXR<br>AAH63827.1|	1e−124	Identities: 216/330 (65%)<br>positives: 260/330 (78%)<br>gaps: 17/330 (5%)
jgi|Triad1|50786|fgeneshTA2_pm.<br>C_scaffold_12000032	HNF4<br>NP_849180.1	1e−125	Identities: 223/324 (68%)<br>positives: 265/324 (81%)<br>aps: 7/324 (2%)
>jgi|Triad1|9010|gw1.23.150.1<br>DNA-binding domain	Human ERRγ<br>AAH64700.1	1e−28	Identities: 53/85 (62%)<br>positives: 67/85 (78%)<br>gaps: 0/85 (0%)
>jgi|Triad1|21656|e_gw1.2.1246.1	Human COUP<br>ref|NP_005645.1	3e−73	Identities: 143/322 (44%)<br>positives; 205/322 (63%)<br>gaps; 12/322 (3%)

The *Trichoplax* genome at JGI was searched with the amino acid sequence for human ERα.
Column 1 lists the five entries that were retrieved, two of which correspond to the ligand-binding and DNA-binding domains of human ERRγ.
Columns 2 and 3 show the highest scoring entry in GenBank and its BLAST score.
Column 4 shows the % identities, positives, which include identities and conservative replacements, and the gaps in the BLAST alignment.

binding domain on vertebrate ERs, with the goal of determining how similar each invertebrate ER is to the steroid-binding vertebrate ERs. To our surprise, the *Capitella* ER is closest to vertebrate ERβ, unlike the other invertebrate ER sequences, which are closest to ERα. For example, BLAST found minnow ERβ [GenBank Accession No. ABS84945] as the closest vertebrate protein to *Capitella* ER. Following in the BLAST output were eleven ERβ entries and then amphioxus ERR [GenBank Accession No. AAU88063]. Much later in the BLAST output was mouse ERα [GenBank Accession No. P19785]. BLAST searches showed that the other invertebrate ERs were closest to vertebrate ERα, and then closest to vertebrate ERβ and then closest to vertebrate ERR.

Table 2
Invertebrate estrogen receptors and estrogen related receptors in JGI genomes and GenBank

Animal	Characteristics	ER	ERR
Trichoplax	Diploblast Placozoan	No	Yes
Fruit Fly *Drosophila*	Ecdysozoan Arthropod	No	Yes
Water Flea *Daphnia*	Ecdysozoan Arthropod	No	Yes
Sea Slug *Aplysia*[b]	Lophotrochozoan Mollusk	Yes[a]	None in GenBank
Snail *Thais*[b]	Lophotrochozoan Mollusk	Yes[a]	None in GenBank
Snail *Marissa*[b]	Lophotrochozoan Mollusk	Yes[a]	Yes
Oyster[b] *Crassostrea*	Lophotrochozoan Mollusk	Yes[a]	None in GenBank
Octopus[b]	Lophotrochozoan Mollusk	Yes[a]	None in GenBank
Snail *Lottia*	Lophotrochozoan Mollusk	Yes	No
Bristle Worm *Capitella*	Lophotrochozoan Annelid	No	Yes
Leech *Helobdella*	Lophotrochozoan Annelid	No	Yes

[a] Constitutive transcriptional activity. Receptor does not bind estradiol.
[b] Complete genome has not been sequenced.

To follow-up these BLAST analyses, we did pairwise BLAST comparisons of each invertebrate ER with human ERα, ERβ, and ERRγ. As shown in Table 2, pairwise BLAST analyses show that *Capitella* ER is closer to human ERβ than to human ERα. Interestingly, octopus ER has about equal sequence similarity to human ERα and human ERβ. However, a BLAST search of GenBank found that octopus ER clearly was closest to ERα. BLAST found the closest vertebrate sequence to octopus ER was golden hamster ERα [GenBank Accession No. AAD53956], which was followed by over twenty ERα sequences. The pairwise BLAST analyses in Table 2 show that the other invertebrate ERs are closer to ERα than to ERβ.

Invertebrate ERs evolved in lophotrochozoans

To clarify further the evolutionary relationships of various invertebrate and vertebrate ERRs and ERs, we constructed a phylogenetic tree of their ligand-binding domains as shown in Fig. 1. The vertebrate ER and ERR part of the phylogeny is in agreement with previous analyses [8,29,39]. The phylogeny indicates that vertebrate ERs and invertebrate ERs diverged from a common ancestor at node A before the evolution of deuterostomes.

Like our BLAST analyses [Table 2], the phylogeny shows that *Capitella* ER has diverged substantially from the other invertebrate ERs, which cluster together.

The phylogeny and the absence of invertebrate ERs in ecdyzoa suggests that invertebrate ERs arose in a lophotrochozoan from an ERR-like ancestor. A practical application of this phylogeny is to suggest that invertebrate ERs are likely to have function(s) that resemble ERR functions [2,17–20].

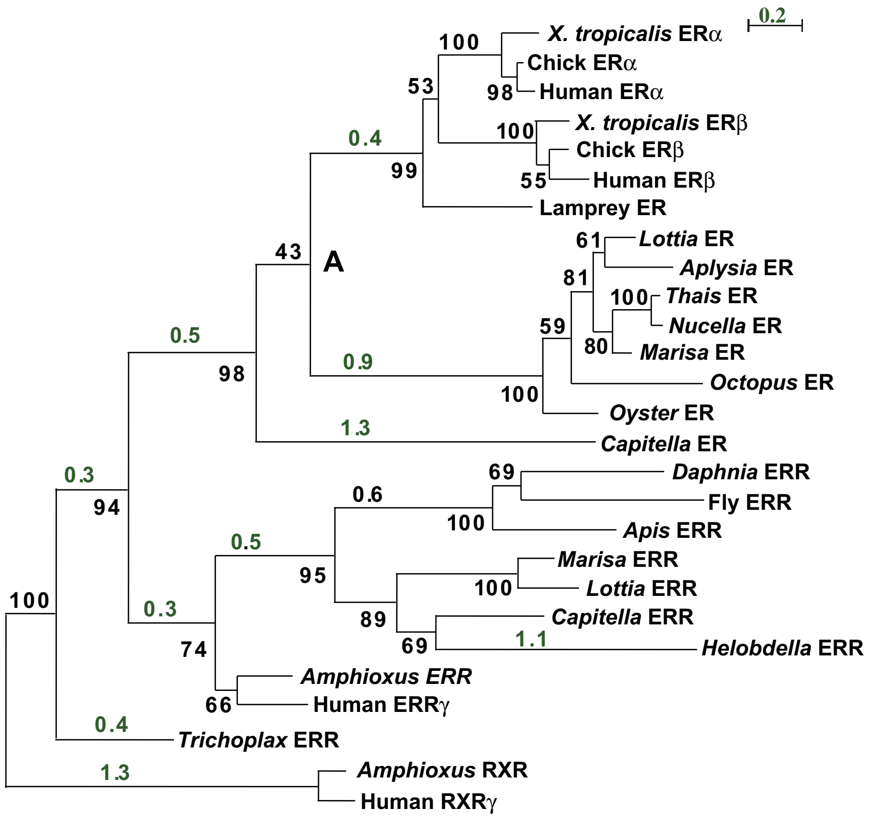

Fig. 1. Phylogenetic analysis of invertebrate and vertebrate ERs and ERRs. The phylogenetic tree was constructed using PhyML [36] under the WAG substitution model [38], with a gamma distribution of rates between sites (four categories, parameter α estimated by PhyML), and 100 bootstrap replicates. Shown at the nodes are bootstrap values for each branch of the tree, which is the percent this cluster was found in the 100 bootstrap trials. Branches with bootstrap values that are greater than fifty percent are significant. Branch lengths are proportional to the distance between proteins. Due to space limitations, we show values for selected branches. Human RXRα and amphioxus RXR were used as outgroups for the phylogenetic tree.

The evolution of invertebrate ERs from an ERR-like ancestor is consistent with functional similarities between invertebrate ERs and vertebrate ERRs. Both vertebrate ERRs and invertebrate ERs are constitutively active and do not bind estradiol. The crystal structures of human ERRs [11–14] and a 3D model of octopus ER [40] indicates that their ligand-binding domains are too small to accommodate estradiol.

Steroid-regulated vertebrate ERs evolved in a deuterostome

The absence of an invertebrate ER outside of lophotrochozoans and the absence of an invertebrate ER in the recently completed sea urchin genome [41] suggests that a steroid-binding vertebrate ER evolved in a deuterostome [27,28,42,43], in which case, vertebrate and invertebrate ERs evolved from different ancestors.

Which mutations led to the evolution of estrogen-dependent activation in vertebrate ERs? Analyses of the 3D structures of human ERRα [11] and ERRγ [13] reveals that the volume of their ligand-binding pockets are about 100 and 220 $Å^3$, respectively, which is much less than 369 $Å^3$ found in human ERα [14]. The more compact ligand-binding pocket in ERRs [13,14] is thought to explain why ERRs do not bind estradiol, which has a van der Waals volume of 251 $Å^3$ [44] and, thus, easily fits into human ERα. The 3D structures of ERRα and ERRγ also reveal that the AF2 domain is in a position to have productive interactions with coactivators and regulate gene transcription.

Information clarifying the basis for the transcriptional properties of ERRγ comes from Greschik et al. [13], who modeled estradiol in ERRγ and compared it with estradiol in ERα [45]. They identified two residues, Leu-345 and Phe-435 in ERRγ that had steric clashes with the D ring of estradiol. Mutation of these residues to Ile and Leu, respectively, as found in ERα, reduced steric interference with estradiol. As a result, the mutant ERRγ bound estradiol, although with low affinity. However, as Greschik et al. [13] noted, unexpectedly, there was no change in transcriptional activity of the ERRγ mutant, which suggests that in the estradiol-ERRγ mutant complex, the AF2 domain is in the proper configuration to bind coactivators. This contrasts with binding of estradiol to vertebrate ERs, which causes a conformational change in AF2 on α-helix 12, so that the ER can bind co-activators [45]. A similar conformational change occurs in other steroid receptors upon binding of their cognate steroid [15,16].

If the ligand-activated vertebrate ER evolved from an ERR, then Greschik et al.'s studies indicate that additional mutation(s) in the ERR ancestor had to occur to increase the affinity for estradiol and also alter the configuration of AF2 in order for binding of a ligand to be required for transcriptional activity. Alignment ERRγ with human ERα [13,40] reveals an insertion of a total of twelve amino acids distributed among three sites in the steroid-binding domain of ERα compared to ERRγ. These insertions map to loops between α-helices in human ERα. One or more of these insertions may be important in altering the ligand-binding pocket and/or conformation of AF2 to yield a steroid-dependent ER.

References

[1] V. Giguere, N. Yang, P. Segui, R.M. Evans, Identification of a new class of steroid hormone receptors, Nature 331 (1988) 91–94.
[2] V. Giguere, To ERR in the estrogen pathway, Trends Endocrinol. Metab. 13 (2002) 220–225.
[3] B. Horard, J.M. Vanacker, Estrogen receptor-related receptors: orphan receptors desperately seeking a ligand, J. Mol. Endocrinol. 31 (2003) 349–357.
[4] H. Hong, L. Yang, M.R. Stallcup, Hormone-independent transcriptional activation and coactivator binding by novel orphan nuclear receptor ERR3, J. Biol. Chem. 274 (1999) 22618–22626.
[5] S.A. Kliewer, J.M. Lehmann, T.M. Willson, Orphan nuclear receptors: shifting endocrinology into reverse, Science 284 (1999) 757–760.
[6] J.A. Gustafsson, Seeking ligands for lonely orphan receptors, Science 284 (1999) 1285–1286.
[7] M.E. Baker, Steroid receptor phylogeny and vertebrate origins, Mol. Cell. Endocrinol. 135 (1997) 101–107.
[8] H. Escriva, F. Delaunay, V. Laudet, Ligand binding and nuclear receptor evolution, Bioessays 22 (2000) 717–727.
[9] S. Bertrand, F.G. Brunet, H. Escriva, G. Parmentier, V. Laudet, M. Robinson-Rechavi, Evolutionary genomics of nuclear receptors: from twenty-five ancestral genes to derived endocrine systems, Mol. Biol. Evol. 21 (2004) 1923–1937.
[10] J.M. Olefsky, Nuclear receptor minireview series, J. Biol. Chem. 276 (2001) 36863–36864.
[11] J. Kallen, J.M. Schlaeppi, F. Bitsch, I. Filipuzzi, A. Schilb, V. Riou, A. Graham, A. Strauss, M. Geiser, B. Fournier, Evidence for ligand-independent transcriptional activation of the human estrogen-related receptor alpha (ERRalpha): crystal structure of ERRalpha ligand binding domain in complex with peroxisome proliferator-activated receptor coactivator-1alpha, J. Biol. Chem. 279 (2004) 49330–49337.
[12] J. Kallen, R. Lattmann, R. Beerli, A. Blechschmidt, M.J. Blommers, M. Geiser, J. Ottl, J.M. Schlaeppi, A. Strauss, B. Fournier, Crystal structure of human estrogen-related receptor alpha in complex with a synthetic inverse agonist reveals its novel molecular mechanism, J. Biol. Chem. 282 (2007) 23231–23239.
[13] H. Greschik, J.M. Wurtz, S. Sanglier, W. Bourguet, A. van Dorsselaer, D. Moras, J.P. Renaud, Structural and functional evidence for ligand-independent transcriptional activation by the estrogen-related receptor 3, Mol. Cell 9 (2002) 303–313.
[14] G. Benoit, M. Malewicz, T. Perlmann, Digging deep into the pockets of orphan nuclear receptors: insights from structural studies, Trends Cell Biol. 14 (2004) 369–376.
[15] M.G. Rosenfeld, C.K. Glass, Coregulator codes of transcriptional regulation by nuclear receptors, J. Biol. Chem. 276 (2001) 36865–36868.
[16] D.M. Lonard, B.W. O'Malley, Expanding functional diversity of the coactivators, Trends Biochem. Sci. 30 (2005) 126–132.
[17] P.L. Bardet, V. Laudet, J.M. Vanacker, Studying non-mammalian models? Not a fool's ERRand!, Trends Endocrinol Metab. 17 (2006) 166–171.
[18] A.M. Tremblay, V. Giguere, The NR3B subgroup: an ovERRview, Nucl. Recept. Signal. 5 (2007) e009.
[19] W.A. Alaynick, R.P. Kondo, W. Xie, W. He, C.R. Dufour, M. Downes, J.W. Jonker, W. Giles, R.K. Naviaux, V. Giguere, R.M. Evans, ERRgamma directs and maintains the transition to oxidative metabolism in the postnatal heart, Cell Metab. 6 (2007) 13–24.
[20] J.A. Villena, M.B. Hock, W.Y. Chang, J.E. Barcas, V. Giguere, A. Kralli, Orphan nuclear receptor estrogen-related receptor alpha is essential for adaptive thermogenesis, Proc. Natl. Acad. Sci. USA 104 (2007) 1418–1423.
[21] J. Keay, J.T. Bridgham, J.W. Thornton, The octopus vulgaris estrogen receptor is a constitutive transcriptional activator: evolutionary and functional implications, Endocrinology 147 (2006) 3861–3869.
[22] R. Bannister, N. Beresford, D. May, E.J. Routledge, S. Jobling, M. Rand-Weaver, Novel estrogen receptor-related Transcripts in Marisa coronaritis; a freshwater snail with reported sensitivity to estrogenic chemicals, Environ. Sci. Technol. 41 (2007) 2643–2650.
[23] M. Kajiwara, S. Kuraku, T. Kurokawa, K. Kato, S. Toda, H. Hirose, S. Takahashi, Y. Shibata, T. Iguchi, T. Matsumoto, T. Miyata, T. Miura, Y. Takahashi, Tissue preferential expression of estrogen receptor gene in the marine snail, Thais clavigera, Gen. Comp. Endocrinol. 148 (2006) 315–326.
[24] T. Matsumoto, A.M. Nakamura, K. Mori, I. Akiyama, H. Hirose, Y. Takahashi, Oyster estrogen receptor: cDNA cloning and immunolocalization, Gen. Comp. Endocrinol. 151 (2007) 195–201.
[25] J.W. Thornton, E. Need, D. Crews, Resurrecting the ancestral steroid receptor: ancient origin of estrogen signaling, Science 301 (2003) 1714–1717.
[26] G.G. Kuiper, B. Carlsson, K. Grandien, E. Enmark, J. Haggblad, S. Nilsson, J.A. Gustafsson, Comparison of the ligand binding specificity and transcript tissue distribution of estrogen receptors alpha and beta, Endocrinology 138 (1997) 863–870.
[27] M.E. Baker, Amphioxus, a primitive chordate, is on steroids: evidence for sex steroids and steroidogenic enzymes, Endocrinology 148 (2007) 3551–3553.
[28] M.E. Baker, Evolution of adrenal and sex steroid action in vertebrates: a ligand-based mechanism for complexity, Bioessays 25 (2003) 396–400.
[29] J.W. Thornton, Evolution of vertebrate steroid receptors from an ancestral estrogen receptor by ligand exploitation and serial genome expansions, Proc. Natl. Acad. Sci. USA 98 (2001) 5671–5676.
[30] B. Schierwater, My favorite animal, Trichoplax adhaerens, Bioessays 27 (2005) 1294–1302.
[31] B. Schierwater, R. DeSalle, Can we ever identify the urmetazoan?, Integr Comp. Biol. 47 (2007) 670–676.
[32] S.L. Dellaporta, A. Xu, S. Sagasser, W. Jakob, M.A. Moreno, L.W. Buss, B. Schierwater, Mitochondrial genome of Trichoplax adhaerens supports placozoa as the basal lower metazoan phylum, Proc. Natl. Acad. Sci. USA 103 (2006) 8751–8756.
[33] S.F. Altschul, T.L. Madden, A.A. Schaffer, J. Zhang, Z. Zhang, W. Miller, D.J. Lipman, Gapped BLAST and PSI-BLAST: a new generation of protein database search programs, Nucleic Acids Res. 25 (1997) 3389–3402.
[34] M.A. Larkin, G. Blackshields, N.P. Brown, R. Chenna, P.A. McGettigan, H. McWilliam, F. Valentin, I.M. Wallace, A. Wilm, R. Lopez, J.D. Thompson, T.J.

Gibson, D.G. Higgins, Clustal W and Clustal X version 2.0, Bioinformatics 23 (2007) 2947–2948.
[35] N. Saitou, M. Nei, The neighbor-joining method: a new method for reconstructing phylogenetic trees, Mol. Biol. Evol. 4 (1987) 406–425.
[36] S. Guindon, O. Gascuel, A simple, fast, and accurate algorithm to estimate large phylogenies by maximum likelihood, Syst. Biol. 52 (2003) 696–704.
[37] R.C. Edgar, MUSCLE: a multiple sequence alignment method with reduced time and space complexity, BMC Bioinform. 5 (2004) 113.
[38] S. Whelan, N. Goldman, A general empirical model of protein evolution derived from multiple protein families using a maximum-likelihood approach, Mol. Biol. Evol. 18 (2001) 691–699.
[39] M.E. Baker, Adrenal and sex steroid receptor evolution: environmental implications, J. Mol. Endocrinol. 26 (2001) 119–125.
[40] M.E. Baker, C. Chandsawangbhuwana, Analysis of 3D models of octopus estrogen receptor with estradiol: evidence for steric clashes that prevent estrogen binding, Biochem. Biophys. Res. Commun. 361 (2007) 782–788.
[41] M. Howard-Ashby, S.C. Materna, C.T. Brown, L. Chen, R.A. Cameron, E.H. Davidson, Gene families encoding transcription factors expressed in early development of Strongylocentrotus purpuratus, Dev. Biol. 300 (2006) 90–107.
[42] M. Paris, K. Pettersson, M. Schubert, S. Bertrand, I. Pongratz, H. Escriva, V. Laudet, An amphioxus orthologue of the estrogen receptor that does not bind estradiol: insights into estrogen receptor evolution, BMC Evol. Biol. 8 (2008) 219.
[43] N.H. Putnam, T. Butts, D.E. Ferrier, R.F. Furlong, U. Hellsten, T. Kawashima, M. Robinson-Rechavi, E. Shoguchi, A. Terry, J.K. Yu, E.L. Benito-Gutierrez, I. Dubchak, J. Garcia-Fernandez, J.J. Gibson-Brown, I.V. Grigoriev, A.C. Horton, P.J. de Jong, J. Jurka, V.V. Kapitonov, Y. Kohara, Y. Kuroki, E. Lindquist, S. Lucas, K. Osoegawa, L.A. Pennacchio, A.A. Salamov, Y. Satou, T. Sauka-Spengler, J. Schmutz, I.T. Shin, A. Toyoda, M. Bronner-Fraser, A. Fujiyama, L.Z. Holland, P.W. Holland, N. Satoh, D.S. Rokhsar, The amphioxus genome and the evolution of the chordate karyotype, Nature 453 (2008) 1064–1071.
[44] A.A. Bogan, F.E. Cohen, T.S. Scanlan, Natural ligands of nuclear receptors have conserved volumes, Nat. Struct. Biol. 5 (1998) 679–681.
[45] A.M. Brzozowski, A.C. Pike, Z. Dauter, R.E. Hubbard, T. Bonn, O. Engstrom, L. Ohman, G.L. Greene, J.A. Gustafsson, M. Carlquist, Molecular basis of agonism and antagonism in the oestrogen receptor, Nature 389 (1997) 753–758.
