# Supplementary material for: Trichoplax adhaerens reveals a network of nuclear receptors sensitive to 9-cis-retinoic acid at the base of metazoan evolution
Source: PeerJ. 2017 Sep 29;5:e3789. doi: 10.7717/peerj.3789 (PMC5624297; doi:10.7717/peerj.3789)
Supplement: File S2 — ClustalO alignments of HNF4, COUP-TF and ERR sequences from selected species. [file peerj-05-3789-s002.zip › ERR/23.09/ERR sequences.docx]

>TaERR Protein ID 16711

NDVIKKLLEAEPPMLPACPDPEAEDSGIRTITIICEMVERELVMVIDWAKRIPGYTSLCLNDQVVLLQASWLEVFMIDLAFRSMPYDNKLVYACDMVMGHKQSRAAGLDEINRHAFELVTKYRSISMDKQEFACLKAIALVNSDSRNLTDVSRVESVQGTLYLALQRYTLLNYPEQPLRFAQLLMTLPELKAISSRGIEKLFSIKVAGEVPMYNLLLEMLEA

>sp|P62508|ERR3_HUMAN Estrogen-related receptor gamma OS=Homo sapiens GN=ESRRG PE=1 SV=1

MDSVELCLPESFSLHYEEELLCRMSNKDRHIDSSCSSFIKTEPSSPASLTDSVNHHSPGG

SSDASGSYSSTMNGHQNGLDSPPLYPSAPILGGSGPVRKLYDDCSSTIVEDPQTKCEYML

NSMPKRLCLVCGDIASGYHYGVASCEACKAFFKRTIQGNIEYSCPATNECEITKRRRKSC

QACRFMKCLKVGMLKEGVRLDRVRGGRQKYKRRIDAENSPYLNPQLVQPAKKPYNKIVSH

LLVAEPEKIYAMPDPTVPDSDIKALTTLCDLADRELVVIIGWAKHIPGFSTLSLADQMSL

LQSAWMEILILGVVYRSLSFEDELVYADDYIMDEDQSKLAGLLDLNNAILQLVKKYKSMK

LEKEEFVTLKAIALANSDSMHIEDVEAVQKLQDVLHEALQDYEAGQHMEDPRRAGKMLMT

LPLLRQTSTKAVQHFYNIKLEGKVPMHKLFLEMLEAKV

>sp|P11474|ERR1_HUMAN Steroid hormone receptor ERR1 OS=Homo sapiens GN=ESRRA PE=1 SV=3

MSSQVVGIEPLYIKAEPASPDSPKGSSETETEPPVALAPGPAPTRCLPGHKEEEDGEGAG

PGEQGGGKLVLSSLPKRLCLVCGDVASGYHYGVASCEACKAFFKRTIQGSIEYSCPASNE

CEITKRRRKACQACRFTKCLRVGMLKEGVRLDRVRGGRQKYKRRPEVDPLPFPGPFPAGP

LAVAGGPRKTAAPVNALVSHLLVVEPEKLYAMPDPAGPDGHLPAVATLCDLFDREIVVTI

SWAKSIPGFSSLSLSDQMSVLQSVWMEVLVLGVAQRSLPLQDELAFAEDLVLDEEGARAA

GLGELGAALLQLVRRLQALRLEREEYVLLKALALANSDSVHIEDAEAVEQLREALHEALL

EYEAGRAGPGGGAERRRAGRLLLTLPLLRQTAGKVLAHFYGVKLEGKVPMHKLFLEMLEA

MMD

>sp|O95718|ERR2_HUMAN Steroid hormone receptor ERR2 OS=Homo sapiens GN=ESRRB PE=1 SV=2

MSSDDRHLGSSCGSFIKTEPSSPSSGIDALSHHSPSGSSDASGGFGLALGTHANGLDSPP

MFAGAGLGGTPCRKSYEDCASGIMEDSAIKCEYMLNAIPKRLCLVCGDIASGYHYGVASC

EACKAFFKRTIQGNIEYSCPATNECEITKRRRKSCQACRFMKCLKVGMLKEGVRLDRVRG

GRQKYKRRLDSESSPYLSLQISPPAKKPLTKIVSYLLVAEPDKLYAMPPPGMPEGDIKAL

TTLCDLADRELVVIIGWAKHIPGFSSLSLGDQMSLLQSAWMEILILGIVYRSLPYDDKLV

YAEDYIMDEEHSRLAGLLELYRAILQLVRRYKKLKVEKEEFVTLKALALANSDSMYIEDL

EAVQKLQDLLHEALQDYELSQRHEEPWRTGKLLLTLPLLRQTAAKAVQHFYSVKLQGKVP

MHKLFLEMLEAKVGQEQLRGSPKDERMSSHDGKCPFQSAAFTSRDQSNSPGIPNPRPSSP

TPLNERGRQISPSTRTPGGQGKHLWLTM

>tr|A0JM86|A0JM86_XENTR Estrogen-related receptor alpha OS=Xenopus tropicalis GN=esrra PE=2 SV=1

MSSRDRRPDLCIKAEPGTPESIGRRSPSGSSDSSGHGPDPPGQRCCRDEEQDDVSGRGKY

VLNSIPKRLCLVCGDVASGYHYGVASCEACKAFFKRTIQGNIEYSCPASNECEITKRRRK

ACQACRFTKCLRVGMLKEGVRLDRVRGGRQKYKRRPEGEILQYTSGGPAQQASATVVKKQ

TPVNAVVSHLLVAEPDKLFAMPDPALPDGYLKSMSTLCDLADREIVIIISWAKNIPGFSS

LSLSDQMSLLQSVWMEVLLLGVVFRSLPYEDEVVFAEDFVLDEESSRSARLTDLCSCILH

LVRKYRTLRVEKEEYVMLKALTLTNSDSVHIEDPDAVLRLRDALQEALSEYESGRHPEEP

CRDGKLLLTLPLLRQTAGRVLQHFHALREEGAVPMHKLFLEMLEAMMD

>tr|Q9VSE9|Q9VSE9_DROME Estrogen-related receptor OS=Drosophila melanogaster GN=ERR PE=2 SV=1

MSDGVSILHIKQEVDTPSASCFSPSSKSTATQSGTNGLKSSPSVSPERQLCSSTTSLSCD

LHNVSLSNDGDSLKGSGTSGGNGGGGGGGTSGGNATNASAGAGSGSVRDELRRLCLVCGD

VASGFHYGVASCEACKAFFKRTIQGNIEYTCPANNECEINKRRRKACQACRFQKCLLMGM

LKEGVRLDRVRGGRQKYRRNPVSNSYQTMQLLYQSNTTSLCDVKILEVLNSYEPDALSVQ

TPPPQVHTTSITNDEASSSSGSIKLESSVVTPNGTCIFQNNNNNDPNEILSVLSDIYDKE

LVSVIGWAKQIPGFIDLPLNDQMKLLQVSWAEILTLQLTFRSLPFNGKLCFATDVWMDEH

LAKECGYTEFYYHCVQIAQRMERISPRREEYYLLKALLLANCDILLDDQSSLRAFRDTIL

NSLNDVVYLLRHSSAVSHQQQLLLLLPSLRQADDILRRFWRGIARDEVITMKKLFLEMLE

PLAR
