# Supplementary material for: Trichoplax adhaerens reveals a network of nuclear receptors sensitive to 9-cis-retinoic acid at the base of metazoan evolution
Source: PeerJ. 2017 Sep 29;5:e3789. doi: 10.7717/peerj.3789 (PMC5624297; doi:10.7717/peerj.3789)
Supplement: File S2 — ClustalO alignments of HNF4, COUP-TF and ERR sequences from selected species. [file peerj-05-3789-s002.zip › HNF4/HNF4 sequences.docx]

>TaHNF4 Protein ID 50786

MVTTCAICGDRATGKHYGAPSCDGCKGFFRRSVRQNHVYTCRFGRSCVIDKDKRNQCRYCRLKKCFRAGMKKEAVQSERDRISRRPSEDQSGGSELTCSTLLAAELLSQPQSSPPRECSLDVIRMASVNDICESMRQQLLLLVEWAKYIPSFCELLLDDQVTLLRAHACEHLMLGVARRSMRLKNILLLGNDLILPRHLPEEPEIARIACRIMDELCLPMLTHNIDDTEYACLKAIVFFNPDAKGLNEPMKIKRLRFQIQLALEDYINDRQYDSRGRFGEMLLLLPNLHSIAMQSVEHLQFARLFGTAKVDSLLQEMLLGGKTSPIM*

>sp|P41235|HNF4A_HUMAN Hepatocyte nuclear factor 4-alpha OS=Homo sapiens GN=HNF4A PE=1 SV=3

MRLSKTLVDMDMADYSAALDPAYTTLEFENVQVLTMGNDTSPSEGTNLNAPNSLGVSALC

AICGDRATGKHYGASSCDGCKGFFRRSVRKNHMYSCRFSRQCVVDKDKRNQCRYCRLKKC

FRAGMKKEAVQNERDRISTRRSSYEDSSLPSINALLQAEVLSRQITSPVSGINGDIRAKK

IASIADVCESMKEQLLVLVEWAKYIPAFCELPLDDQVALLRAHAGEHLLLGATKRSMVFK

DVLLLGNDYIVPRHCPELAEMSRVSIRILDELVLPFQELQIDDNEYAYLKAIIFFDPDAK

GLSDPGKIKRLRSQVQVSLEDYINDRQYDSRGRFGELLLLLPTLQSITWQMIEQIQFIKL

FGMAKIDNLLQEMLLGGSPSDAPHAHHPLHPHLMQEHMGTNVIVANTMPTHLSNGQMCEW

PRPRGQAATPETPQPSPPGGSGSEPYKLLPGAVATIVKPLSAIPQPTITKQEVI

>sp|Q91766|HNF4A_XENLA Hepatocyte nuclear factor 4-alpha OS=Xenopus laevis GN=hnf4a PE=2 SV=2

MRLSKALIDMDMADYTEALDPAYTTLEFENMQVLSIGTDTSTSDVTSLSASNSIGINSLC

AICGDRATGKHYGASSCDGCKGFFRRSVRKNHMYSCRFSRQCVVDKDKRNQCRYCRLKKC

FRAGMKKEAVQNERDRISTRRSSYEDSSLPSINVLIQAEVLSQQITSSVGVLNTDIRGKK

IACIIDVCDSMKQQLLVLVEWAKYIPAFCELPLDDQVALLRAHAGEHLLLGATKRSMMFK

DILLLGNDRLIPRNCPELEVGRVAVRILDELVLPFQELQIDDNEYACLKAIIFFDPDAKG

LSDPTKIKRMRYQVQVSLEDYINDRQYDSRGRFGELLLLLPTLQSITWQMIEQIQFVKLF

GMAKIDNLLQEMLLGGSANEASHTHHHLHPHLVQDHLATNVIVANNTLPSQLHNGQMSTP

ETPQPSPPAGSGAEQYKIVHGTIASINKQPTSIPQSTITKQEAM

>sp|P49866|HNF4_DROME Transcription factor HNF-4 homolog OS=Drosophila melanogaster GN=Hnf4 PE=1 SV=3

MMKHPQDLSVTDDQQLMKVNKVEKMEQELHDPESESHIMHADALASAYPAASQPHSPIGL

ALSPNGGGLGLSNSSNQSSENFALCNGNGNAGSAGGGSASSGSNNNNSMFSPNNNLSGSG

SGTNSSQQQLQQQQQQQSPTVCAICGDRATGKHYGASSCDGCKGFFRRSVRKNHQYTCRF

ARNCVVDKDKRNQCRYCRLRKCFKAGMKKEAVQNERDRISCRRTSNDDPDPGNGLSVISL

VKAENESRQSKAGAAMEPNINEDLSNKQFASINDVCESMKQQLLTLVEWAKQIPAFNELQ

LDDQVALLRAHAGEHLLLGLSRRSMHLKDVLLLSNNCVITRHCPDPLVSPNLDISRIGAR

IIDELVTVMKDVGIDDTEFACIKALVFFDPNAKGLNEPHRIKSLRHQILNNLEDYISDRQ

YESRGRFGEILLILPVLQSITWQMIEQIQFAKIFGVAHIDSLLQEMLLGGELADNPLPLS

PPNQSNDYQSPTHTGNMEGGNQVNSSLDSLATSGGPGSHSLDLEVQHIQALIEANSADDS

FRAYAASTAAAAAAAVSSSSSAPASVAPASISPPLNSPKSQHQHQQHATHQQQQESSYLD

MPVKHYNGSRSGPLPTQHSPQRMHPYQRAVASPVEVSSGGGGLGLRNPADITLNEYNRSE

GSSAEELLRRTPLKIRAPEMLTAPAGYGTEPCRMTLKQEPETGY

>sp|Q91766|HNF4A_XENLA Hepatocyte nuclear factor 4-alpha OS=Xenopus laevis GN=hnf4a PE=2 SV=2

MRLSKALIDMDMADYTEALDPAYTTLEFENMQVLSIGTDTSTSDVTSLSASNSIGINSLC

AICGDRATGKHYGASSCDGCKGFFRRSVRKNHMYSCRFSRQCVVDKDKRNQCRYCRLKKC

FRAGMKKEAVQNERDRISTRRSSYEDSSLPSINVLIQAEVLSQQITSSVGVLNTDIRGKK

IACIIDVCDSMKQQLLVLVEWAKYIPAFCELPLDDQVALLRAHAGEHLLLGATKRSMMFK

DILLLGNDRLIPRNCPELEVGRVAVRILDELVLPFQELQIDDNEYACLKAIIFFDPDAKG

LSDPTKIKRMRYQVQVSLEDYINDRQYDSRGRFGELLLLLPTLQSITWQMIEQIQFVKLF

GMAKIDNLLQEMLLGGSANEASHTHHHLHPHLVQDHLATNVIVANNTLPSQLHNGQMSTP

ETPQPSPPAGSGAEQYKIVHGTIASINKQPTSIPQSTITKQEAM
