# Supplementary material for: Trichoplax adhaerens reveals a network of nuclear receptors sensitive to 9-cis-retinoic acid at the base of metazoan evolution
Source: PeerJ. 2017 Sep 29;5:e3789. doi: 10.7717/peerj.3789 (PMC5624297; doi:10.7717/peerj.3789)
Supplement: File S3 [file peerj-05-3789-s003.docx]

**Supplementary file S2. Identification of *T. adhaerens* predicted L-malate-NADP+ oxidoreductase).**

Blastp result queried against sp|P48163|MAOX_HUMAN NADP-dependent malic enzyme OS=Homo sapiens GN=ME1 PE=1 SV=1

hypothetical protein TRIADDRAFT_50795 [Trichoplax adhaerens]

Sequence ID: [gi|196012437|XP_002116081.1](https://www.ncbi.nlm.nih.gov/protein/196012437?report=genbank&log$=protalign&blast_rank=1&RID=YJG0AZPW01R)Length: 525Number of Matches: 1

[**See 1 more title(s)**](https://blast.ncbi.nlm.nih.gov/Blast.cgi)

hypothetical protein TRIADDRAFT_50795 [Trichoplax adhaerens]

Sequence ID: [gi|190581404|EDV21481.1](https://www.ncbi.nlm.nih.gov/protein/190581404?report=genbank&log$=protalign&blast_rank=1&RID=YJG0AZPW01R)Length: 525Number of Matches:

Related Information

[Gene](https://www.ncbi.nlm.nih.gov/gene?term=196012437%5bPUID%5d%20OR%20190581404%5bPUID%5d&RID=YJG0AZPW01R&log$=genealign&blast_rank=1)-associated gene details

[Identical Proteins](https://www.ncbi.nlm.nih.gov/protein/196012437?report=ipg)-Identical proteins to XP_002116081.1

Range 1: 1 to 523[GenPept](https://www.ncbi.nlm.nih.gov/protein/196012437?report=genbank&log$=protalign&blast_rank=1&RID=YJG0AZPW01R&from=1&to=523)[Graphics](https://www.ncbi.nlm.nih.gov/protein/196012437?report=graph&rid=YJG0AZPW01R%5b196012437%5d&tracks=%5bkey:sequence_track,name:Sequence,display_name:Sequence,id:STD1,category:Sequence,annots:Sequence,ShowLabel:true%5d%5bkey:gene_model_track,CDSProductFeats:false%5d%5bkey:alignment_track,name:other%20alignments,annots:NG%20Alignments|Refseq%20Alignments|Gnomon%20Alignments|Unnamed,shown:false%5d&v=0:549&appname=ncbiblast&link_loc=fromHSP) Next Match Previous Match [First Match](https://blast.ncbi.nlm.nih.gov/Blast.cgi#hsp196012437_1)

| Alignment statistics for match #1 | | | | | | |
| --- | --- | --- | --- | --- | --- | --- |
| **Score** | **Expect** | **Method** | **Identities** | **Positives** | **Gaps** | **Frame** |
| 602 bits(1553) | 0.0() |  | 303/535(57%) | 397/535(74%) | 14/535(2%) |  |

Features:

Query 28 LAFTLEERQQLNIHGLLPPSFNSQEIQVLRVVKNFEHLNSDFDRYLLLMDLQDRNEKLFY 87

+AFTL+ERQ + IHGLLPP+ +QE+QV RV+ + + +D +R++ LM LQDRNEKLFY

Sbjct 1 MAFTLKERQLIGIHGLLPPTVLTQEVQVQRVMNSIKVKPNDLERFIYLMSLQDRNEKLFY 60

Query 88 RVLTSDIEKFMPIVYTPTVGLACQQYSLVFRKPRGLFITIHDRGHIASVLNAWPEDVIKA 147

RV+ +E+ MPI+YTP VGLA GLFI+I+D+G++ S+L +WPE +KA

Sbjct 61 RVIQDHLEELMPIIYTPVVGLA------------GLFISIYDKGNVKSILRSWPEIDVKA 108

Query 148 IVVTDGERILGLGDLGCNGMGIPVGKLALYTACGGMNPQECLPVILDVGTENEELLKDPL 207

IV TDGERILGLGDLGC GMGIPVGKL+LYTAC G+ P +CLPV+LDVGT+N+ELL DP

Sbjct 109 IVFTDGERILGLGDLGCYGMGIPVGKLSLYTACAGVKPSQCLPVMLDVGTDNKELLDDPF 168

Query 208 YIGLRQRRVRGSEYDDFLDEFMEAVSSKYGMNCLIQFEDFANVNAFRLLNKYRNQYCTFN 267

YIG+R+ R RG YD+ ++EFM+AV +YG L+QFEDF N NAFRLL+K+RN++CTFN

Sbjct 169 YIGIRRPRERGPAYDELIEEFMQAVVERYGETTLMQFEDFGNRNAFRLLDKFRNRFCTFN 228

Query 268 DDIQGTasvavagllaalRITKNKLSDQTILFQGAGEAALGIAHLIVMALEKEGLPKEKA 327

DDIQGTA V VAGLL A+RITK L+D LFQGAGEAA+GIA+LIV AL +EGL + A

Sbjct 229 DDIQGTAGVTVAGLLGAMRITKKSLADNVFLFQGAGEAAIGIANLIVSALMEEGLAESDA 288

Query 328 IKKIWLVDSKGLIVKGRA--SLTQEKEKFAHEHEEMKNLEAIVQEIKPTALIGVAAIGGA 385

KK+WLVDSKGL+V R+ + + K+ FAH+H+ + L V+ IKPTA+IGVAAI A

Sbjct 289 RKKVWLVDSKGLVVNNRSKGGINEHKQPFAHDHKYIPTLAEAVKVIKPTAIIGVAAISRA 348

Query 386 FSEQILKDMAAFNERPIIFALSNPTSKAECSAEQCYKITKGRAIFASGSPFDPVTLPNGQ 445

F+++I++DMA NE PIIFALSNPT+K+EC+ E+ Y+ T G+ IFASGSPF P+T +G

Sbjct 349 FTKEIIEDMARINENPIIFALSNPTAKSECTFEEAYQYTNGKCIFASGSPFPPITKKDGS 408

Query 446 TLYPGQGNNSYVFPGVALGVVACGLRQITDNIFLTTAEVIAQQVSDKHLEEGRLYPPLNT 505

+ PGQGNN+Y+FPGVALGV+A + I ++ FL A+ +A QV+D+ L++GR+YPPL

Sbjct 409 QIIPGQGNNAYIFPGVALGVIAAASKTIPESTFLIAAKSLANQVTDEDLKQGRVYPPLTD 468

Query 506 IRDVSLKIAEKIVKDAYQEKTATVYPEPQNKEAFVRSQMYSTDYDQILPDCYSWP 560

IR VS KIA + +Y + P P N + S +YSTDY+ +P Y WP

Sbjct 469 IRKVSRKIAIDVADYSYNIHLSNRLPRPANTAELIDSFIYSTDYESFVPHTYEWP 523

| **Description** | [**Max score**](https://blast.ncbi.nlm.nih.gov/Blast.cgi?CMD=Get&ALIGNMENTS=100&ALIGNMENT_VIEW=Pairwise&CDD_RID=data_cache_seq:495123&CDD_SEARCH_STATE=4&DATABASE_SORT=0&DESCRIPTIONS=100&DYNAMIC_FORMAT=on&ENTREZ_QUERY=txid10228%20%5bORGN%5d&FIRST_QUERY_NUM=0&FORMAT_OBJECT=Alignment&FORMAT_PAGE_TARGET=&FORMAT_TYPE=HTML&GET_SEQUENCE=yes&I_THRESH=&LINE_LENGTH=60&MASK_CHAR=2&MASK_COLOR=1&NCBI_GI=yes&NEW_VIEW=yes&NUM_OVERVIEW=100&OLD_BLAST=false&PAGE=Proteins&QUERY_INDEX=0&QUERY_NUMBER=0&RESULTS_PAGE_TARGET=&RID=YJG0AZPW01R&SHOW_LINKOUT=yes&SHOW_OVERVIEW=yes&STEP_NUMBER=&WORD_SIZE=6&OLD_VIEW=false&DISPLAY_SORT=1&HSP_SORT=1) | [**Total score**](https://blast.ncbi.nlm.nih.gov/Blast.cgi?CMD=Get&ALIGNMENTS=100&ALIGNMENT_VIEW=Pairwise&CDD_RID=data_cache_seq:495123&CDD_SEARCH_STATE=4&DATABASE_SORT=0&DESCRIPTIONS=100&DYNAMIC_FORMAT=on&ENTREZ_QUERY=txid10228%20%5bORGN%5d&FIRST_QUERY_NUM=0&FORMAT_OBJECT=Alignment&FORMAT_PAGE_TARGET=&FORMAT_TYPE=HTML&GET_SEQUENCE=yes&I_THRESH=&LINE_LENGTH=60&MASK_CHAR=2&MASK_COLOR=1&NCBI_GI=yes&NEW_VIEW=yes&NUM_OVERVIEW=100&OLD_BLAST=false&PAGE=Proteins&QUERY_INDEX=0&QUERY_NUMBER=0&RESULTS_PAGE_TARGET=&RID=YJG0AZPW01R&SHOW_LINKOUT=yes&SHOW_OVERVIEW=yes&STEP_NUMBER=&WORD_SIZE=6&OLD_VIEW=false&DISPLAY_SORT=2&HSP_SORT=1) | [**Query cover**](https://blast.ncbi.nlm.nih.gov/Blast.cgi?CMD=Get&ALIGNMENTS=100&ALIGNMENT_VIEW=Pairwise&CDD_RID=data_cache_seq:495123&CDD_SEARCH_STATE=4&DATABASE_SORT=0&DESCRIPTIONS=100&DYNAMIC_FORMAT=on&ENTREZ_QUERY=txid10228%20%5bORGN%5d&FIRST_QUERY_NUM=0&FORMAT_OBJECT=Alignment&FORMAT_PAGE_TARGET=&FORMAT_TYPE=HTML&GET_SEQUENCE=yes&I_THRESH=&LINE_LENGTH=60&MASK_CHAR=2&MASK_COLOR=1&NCBI_GI=yes&NEW_VIEW=yes&NUM_OVERVIEW=100&OLD_BLAST=false&PAGE=Proteins&QUERY_INDEX=0&QUERY_NUMBER=0&RESULTS_PAGE_TARGET=&RID=YJG0AZPW01R&SHOW_LINKOUT=yes&SHOW_OVERVIEW=yes&STEP_NUMBER=&WORD_SIZE=6&OLD_VIEW=false&DISPLAY_SORT=4&HSP_SORT=0) | [**E value**](https://blast.ncbi.nlm.nih.gov/Blast.cgi?CMD=Get&ALIGNMENTS=100&ALIGNMENT_VIEW=Pairwise&CDD_RID=data_cache_seq:495123&CDD_SEARCH_STATE=4&DATABASE_SORT=0&DESCRIPTIONS=100&DYNAMIC_FORMAT=on&ENTREZ_QUERY=txid10228%20%5bORGN%5d&FIRST_QUERY_NUM=0&FORMAT_OBJECT=Alignment&FORMAT_PAGE_TARGET=&FORMAT_TYPE=HTML&GET_SEQUENCE=yes&I_THRESH=&LINE_LENGTH=60&MASK_CHAR=2&MASK_COLOR=1&NCBI_GI=yes&NEW_VIEW=yes&NUM_OVERVIEW=100&OLD_BLAST=false&PAGE=Proteins&QUERY_INDEX=0&QUERY_NUMBER=0&RESULTS_PAGE_TARGET=&RID=YJG0AZPW01R&SHOW_LINKOUT=yes&SHOW_OVERVIEW=yes&STEP_NUMBER=&WORD_SIZE=6&OLD_VIEW=false&DISPLAY_SORT=0&HSP_SORT=0) | [**Ident**](https://blast.ncbi.nlm.nih.gov/Blast.cgi?CMD=Get&ALIGNMENTS=100&ALIGNMENT_VIEW=Pairwise&CDD_RID=data_cache_seq:495123&CDD_SEARCH_STATE=4&DATABASE_SORT=0&DESCRIPTIONS=100&DYNAMIC_FORMAT=on&ENTREZ_QUERY=txid10228%20%5bORGN%5d&FIRST_QUERY_NUM=0&FORMAT_OBJECT=Alignment&FORMAT_PAGE_TARGET=&FORMAT_TYPE=HTML&GET_SEQUENCE=yes&I_THRESH=&LINE_LENGTH=60&MASK_CHAR=2&MASK_COLOR=1&NCBI_GI=yes&NEW_VIEW=yes&NUM_OVERVIEW=100&OLD_BLAST=false&PAGE=Proteins&QUERY_INDEX=0&QUERY_NUMBER=0&RESULTS_PAGE_TARGET=&RID=YJG0AZPW01R&SHOW_LINKOUT=yes&SHOW_OVERVIEW=yes&STEP_NUMBER=&WORD_SIZE=6&DISPLAY_SORT=3&HSP_SORT=3) | **Accession** |  |
| --- | --- | --- | --- | --- | --- | --- | --- |
| 1Select seq ref\|XP_002116081.1\| | [hypothetical protein TRIADDRAFT_50795 [Trichoplax adhaerens]](https://blast.ncbi.nlm.nih.gov/Blast.cgi#alnHdr_196012437) | 602 | 602 | 93% | 0.0 | 57% | [gi\|196012437\|XP_002116081.1](https://www.ncbi.nlm.nih.gov/protein/196012437?report=genbank&log$=prottop&blast_rank=1&RID=YJG0AZPW01R) |

**596bp upstream of ATG**

GTTCATAAACTCCTACTATCCGCAGTCTACCACACTGATAAGTCAAATTTACTGAATTATCAATTTTATATAACCACTAATTCAGCCCAATTGATTTAGTATTACAGTAAGGCTATTATTAAATAGATATTTGTGCAACAGAATGACCATTTGGAGGCTAAATTTTAGCAATAAAAATTAACAAATTACAGATATTTCGGGTCTTACACAAATGCTACATGAAACGAATAGCAGACCTTCCAATAAATGTATTGCACTGAAGTAAACTTCAATGCAGCTTGCTAAGGCATTTATTAGACCTCTTGATATTGGAATTATTAATTGTCGATGTCAAAATTAAATTACTTTAGTCAATACATAACATTCATATACAACTTACATATTATCACCATGTAAAATAAAAAAACTATAGTTAAAAGTTTAGTAAATTCATCATTTAGAATTGTTACCGATTTTGTATAATAGCTAGCATTAGTTTATTTTCGTATATACTCAATTGTTTCATGTTGCATTAACCAGTTTTCGTAATATTTTTGCCCATAGTATATTAATGCAATAATTACATCTACTGTACTTATAAATAGGGAATGGCTTTT

**TRIADDRAFT_50795 transctipt nucleotide sequence**

ATGGCTTTTACTTTGAAAGAGCGCCAGCTGATTGGAATCCATGGTTTGCTACCACCTACAGTATTAACTC

AAGAGGTGCAAGTTCAGCGTGTAATGAATAGTATTAAAGTAAAGCCTAATGATTTAGAGAGATTCATCTA

CTTGATGAGTTTGCAAGATCGAAATGAAAAATTATTTTATCGAGTAATTCAAGATCACCTGGAAGAATTA

ATGCCTATAATCTATACACCTGTTGTCGGTTTAGCGGGTCTTTTTATATCTATTTATGATAAAGGAAACG

TCAAATCGATATTAAGATCTTGGCCAGAAATCGATGTTAAGGCTATTGTATTTACGGATGGTGAACGTAT

TCTAGGTCTAGGAGATCTAGGCTGCTACGGAATGGGAATTCCTGTTGGCAAGCTATCTTTATATACAGCT

TGTGCCGGAGTGAAACCTTCTCAATGCTTACCAGTAATGTTAGATGTTGGAACAGATAATAAGGAATTAC

TCGATGATCCTTTCTACATTGGAATCCGACGTCCACGGGAAAGAGGGCCAGCATATGATGAACTAATTGA

AGAATTTATGCAGGCAGTCGTCGAAAGATATGGTGAAACAACGCTAATGCAATTTGAAGATTTCGGAAAT

AGAAATGCTTTTCGATTACTAGATAAATTTCGAAATCGATTTTGTACTTTTAACGACGACATTCAAGGAA

CTGCTGGGGTTACGGTTGCTGGTTTACTAGGTGCTATGCGAATAACGAAGAAAAGCCTTGCTGATAATGT

ATTCTTGTTTCAAGGAGCTGGAGAAGCTGCTATTGGCATAGCAAACTTGATTGTTAGTGCGTTAATGGAA

GAAGGACTGGCTGAAAGCGATGCTAGAAAGAAAGTATGGCTAGTAGACTCCAAAGGCCTGGTTGTCAATA

ATCGTTCCAAAGGAGGCATTAATGAACATAAGCAGCCATTTGCTCATGATCACAAATACATTCCTACATT

AGCCGAGGCAGTAAAAGTAATAAAACCGACAGCAATTATAGGTGTCGCAGCCATTAGTCGAGCTTTCACT

AAGGAAATTATAGAAGATATGGCAAGAATAAATGAAAATCCAATTATATTTGCTTTAAGTAACCCTACTG

CAAAGTCTGAATGCACCTTTGAAGAGGCCTATCAGTACACTAATGGCAAGTGCATCTTTGCTAGTGGTAG

CCCATTCCCTCCAATTACCAAAAAAGATGGAAGCCAGATTATTCCTGGACAAGGAAATAATGCTTATATA

TTTCCCGGAGTTGCATTAGGAGTCATAGCAGCTGCTTCTAAAACTATCCCTGAATCGACGTTTTTAATTG

CCGCAAAGTCTTTGGCCAATCAAGTTACGGACGAAGACTTGAAACAAGGCAGAGTTTATCCACCATTAAC

AGACATTCGCAAAGTCTCACGTAAAATTGCCATCGATGTCGCGGATTACTCTTACAACATTCATTTATCA

AATCGACTACCCAGACCTGCAAATACAGCTGAATTGATTGACTCATTTATCTACTCCACTGATTATGAGT

CTTTCGTTCCGCATACATACGAATGGCCATCACTATAA

**TaME: TRIADDRAFT_50795 Protein sequence**

MAFTLKERQLIGIHGLLPPTVLTQEVQVQRVMNSIKVKPNDLERFIYLMSLQDRNEKLFYRVIQDHLEELMPIIYTPVVGLAGLFISIYDKGNVKSILRSWPEIDVKAIVFTDGERILGLGDLGCYGMGIPVGKLSLYTACAGVKPSQCLPVMLDVGTDNKELLDDPFYIGIRRPRERGPAYDELIEEFMQAVVERYGETTLMQFEDFGNRNAFRLLDKFRNRFCTFNDDIQGTAGVTVAGLLGAMRITKKSLADNVFLFQGAGEAAIGIANLIVSALMEEGLAESDARKKVWLVDSKGLVVNNRSKGGINEHKQPFAHDHKYIPTLAEAVKVIKPTAIIGVAAISRAFTKEIIEDMARINENPIIFALSNPTAKSECTFEEAYQYTNGKCIFASGSPFPPITKKDGSQIIPGQGNNAYIFPGVALGVIAAASKTIPESTFLIAAKSLANQVTDEDLKQGRVYPPLTDIRKVSRKIAIDVADYSYNIHLSNRLPRPANTAELIDSFIYSTDYESFVPHTYEWP
