# Supplementary figures and images for: Trichoplax adhaerens reveals a network of nuclear receptors sensitive to 9-cis-retinoic acid at the base of metazoan evolution
Source: PeerJ. 2017 Sep 29;5:e3789. doi: 10.7717/peerj.3789 (PMC5624297; doi:10.7717/peerj.3789)

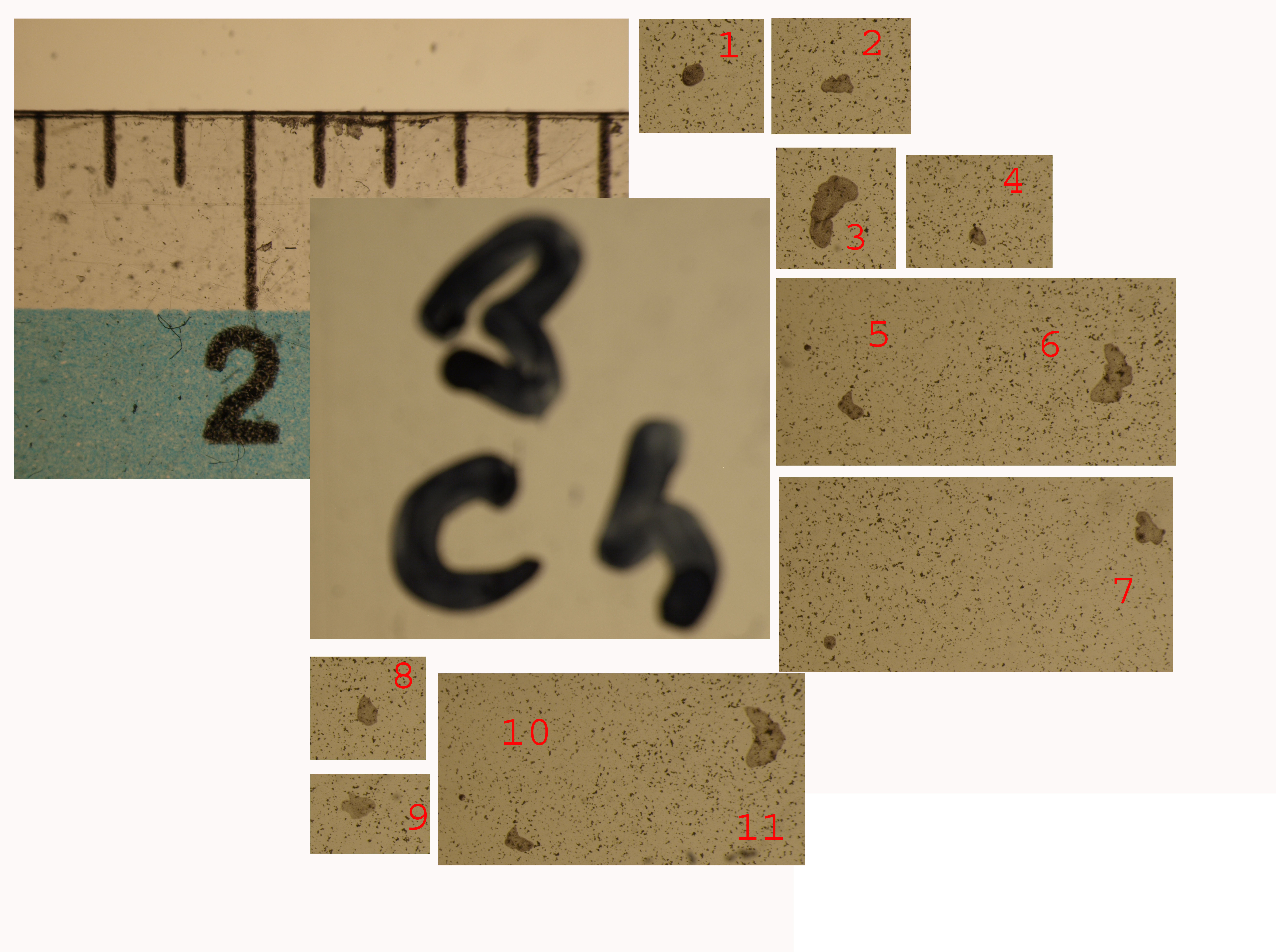

Supplement: File S5 — Images of T. adhaerens that were incubated with specific algal food and copied to one assembled figure which was used for analysis using the ImageJ program. Images were saved in JPG format and divided to two parts in order to comply with maximum space allowed for Supplementary Files. Incubation of T. adhaerens with food composed of all three tested algae was done in duplicate. The second duplicate is provided in Part B. [file peerj-05-3789-s005.zip › File_S5-partA-Compressed Figs-analyzed-saved-in-JPG - Copy/Composed-food-3CH numbered.jpg]

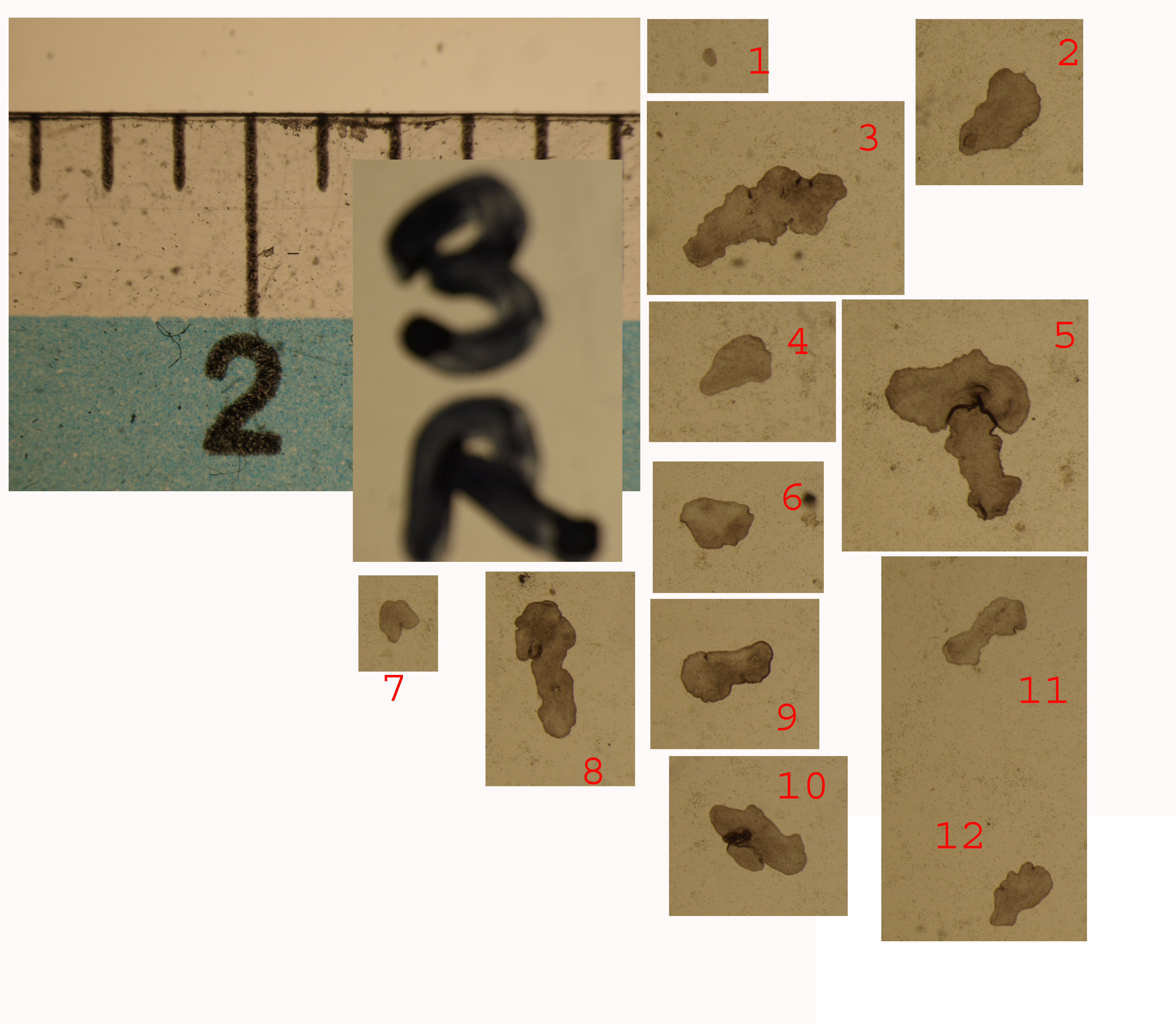

Supplement: File S5 — Images of T. adhaerens that were incubated with specific algal food and copied to one assembled figure which was used for analysis using the ImageJ program. Images were saved in JPG format and divided to two parts in order to comply with maximum space allowed for Supplementary Files. Incubation of T. adhaerens with food composed of all three tested algae was done in duplicate. The second duplicate is provided in Part B. [file peerj-05-3789-s005.zip › File_S5-partA-Compressed Figs-analyzed-saved-in-JPG - Copy/Composed-Food-3R numbered.jpg]

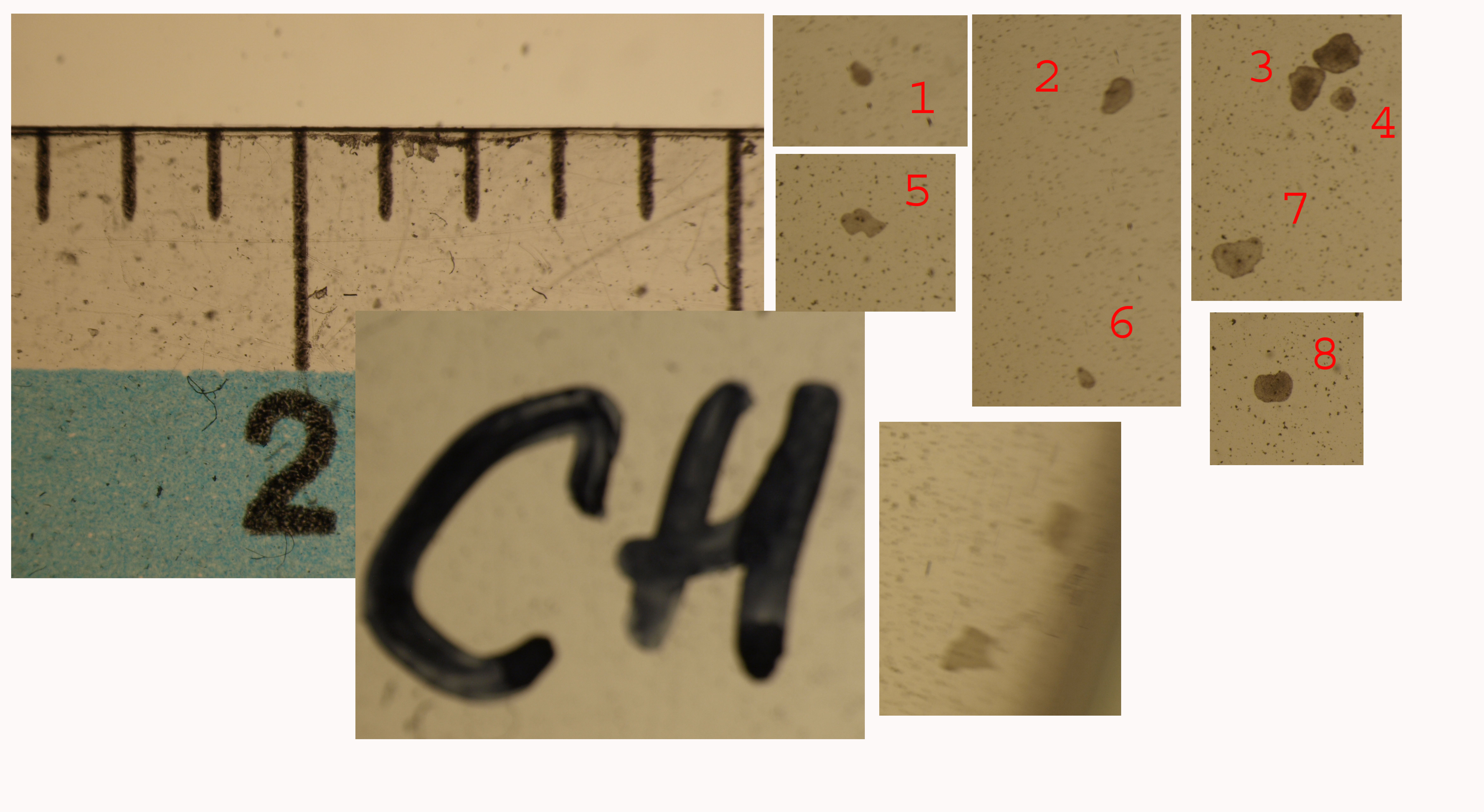

Supplement: File S5 — Images of T. adhaerens that were incubated with specific algal food and copied to one assembled figure which was used for analysis using the ImageJ program. Images were saved in JPG format and divided to two parts in order to comply with maximum space allowed for Supplementary Files. Incubation of T. adhaerens with food composed of all three tested algae was done in duplicate. The second duplicate is provided in Part B. [file peerj-05-3789-s005.zip › File_S5-partA-Compressed Figs-analyzed-saved-in-JPG - Copy/Composed-food-ch numbered.jpg]

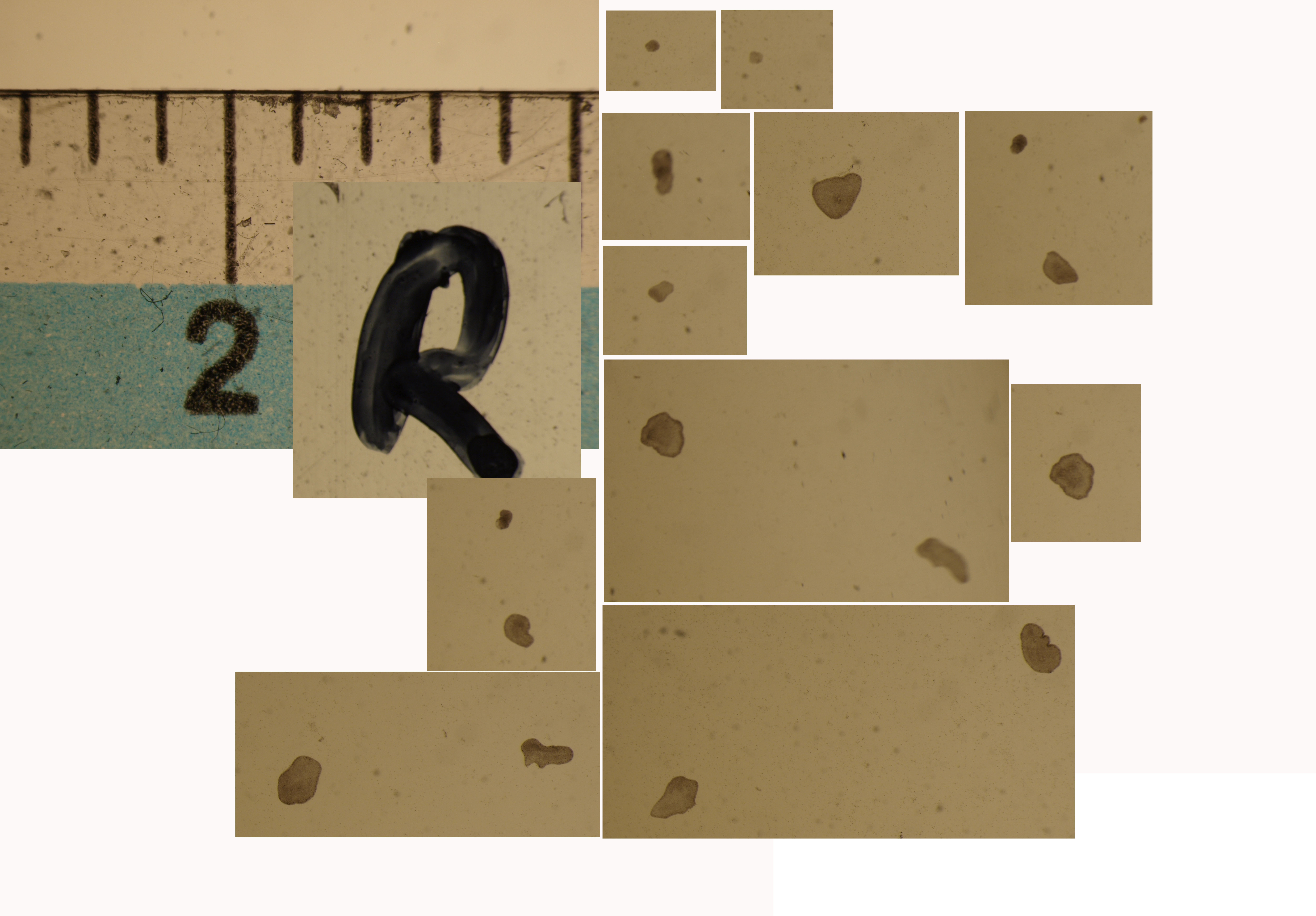

Supplement: File S5 — Images of T. adhaerens that were incubated with specific algal food and copied to one assembled figure which was used for analysis using the ImageJ program. Images were saved in JPG format and divided to two parts in order to comply with maximum space allowed for Supplementary Files. Incubation of T. adhaerens with food composed of all three tested algae was done in duplicate. The second duplicate is provided in Part B. [file peerj-05-3789-s005.zip › File_S5-partA-Compressed Figs-analyzed-saved-in-JPG - Copy/Food-R-composed copy.jpg]

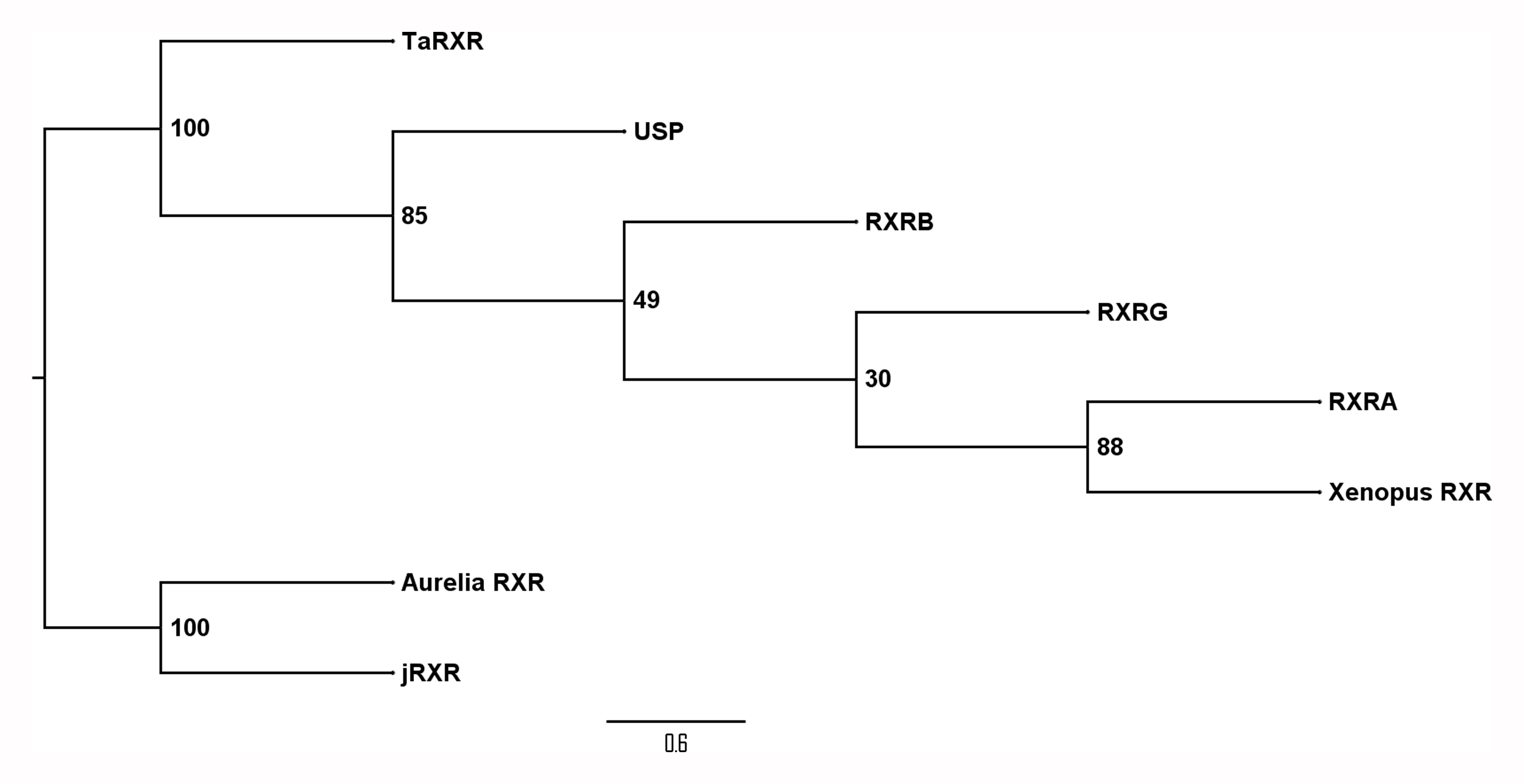

Supplement: Figure S1 — Phylogenetic tree constructed with PhyML algorithm with a 100 bootstrap and SPR distance computation visualized by FigTree of selected metazoan RXR sequences shown in Fig. 1 (and listed in File S1). Bootstrap values are shown next to nodes. [file peerj-05-3789-s009.png]

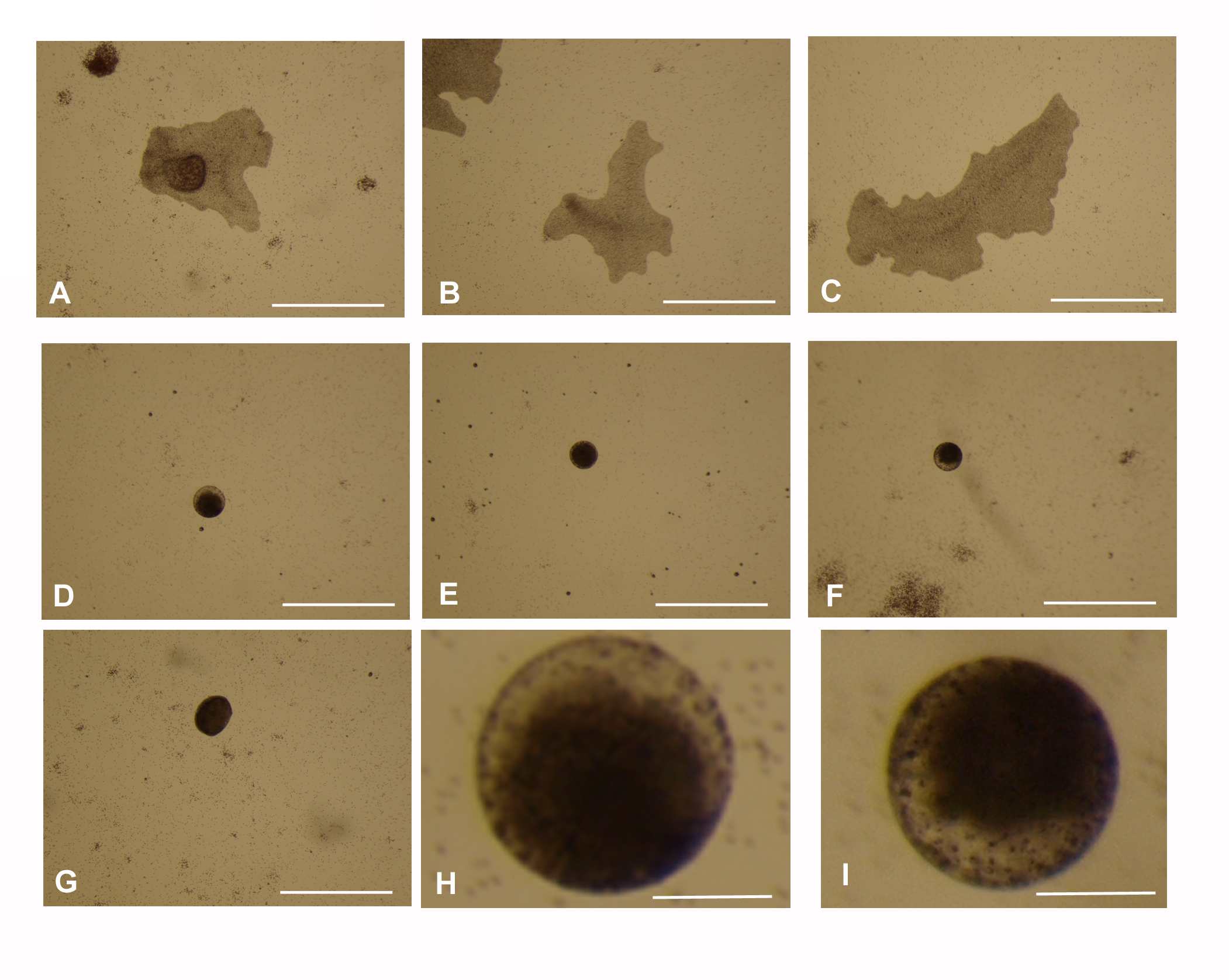

Supplement: Figure S2 — Panels A to C show representative images of three animals from the control group incubated with vehicle and panels D to G show 4 representative animals in the culture treated by 3.3 nM 9-cis-RA for 72 h. Panels H and I show higher magnification of animals from panels D and F. Panel J shows graphical representation of cultures. While the animals treated by 9-cis-RA developed a balloon-like phenotype between point 1 (24 h with 1 animal displaying the balloon-like phenotype) and point 2 (72 h when all animals treated by 9-cis-RA displayed the balloon-like phenotype), the control animals grew and propagated normally. Bars represent 1 mm in panels A to G and 0.1 in panels H and I. [file peerj-05-3789-s010.png]

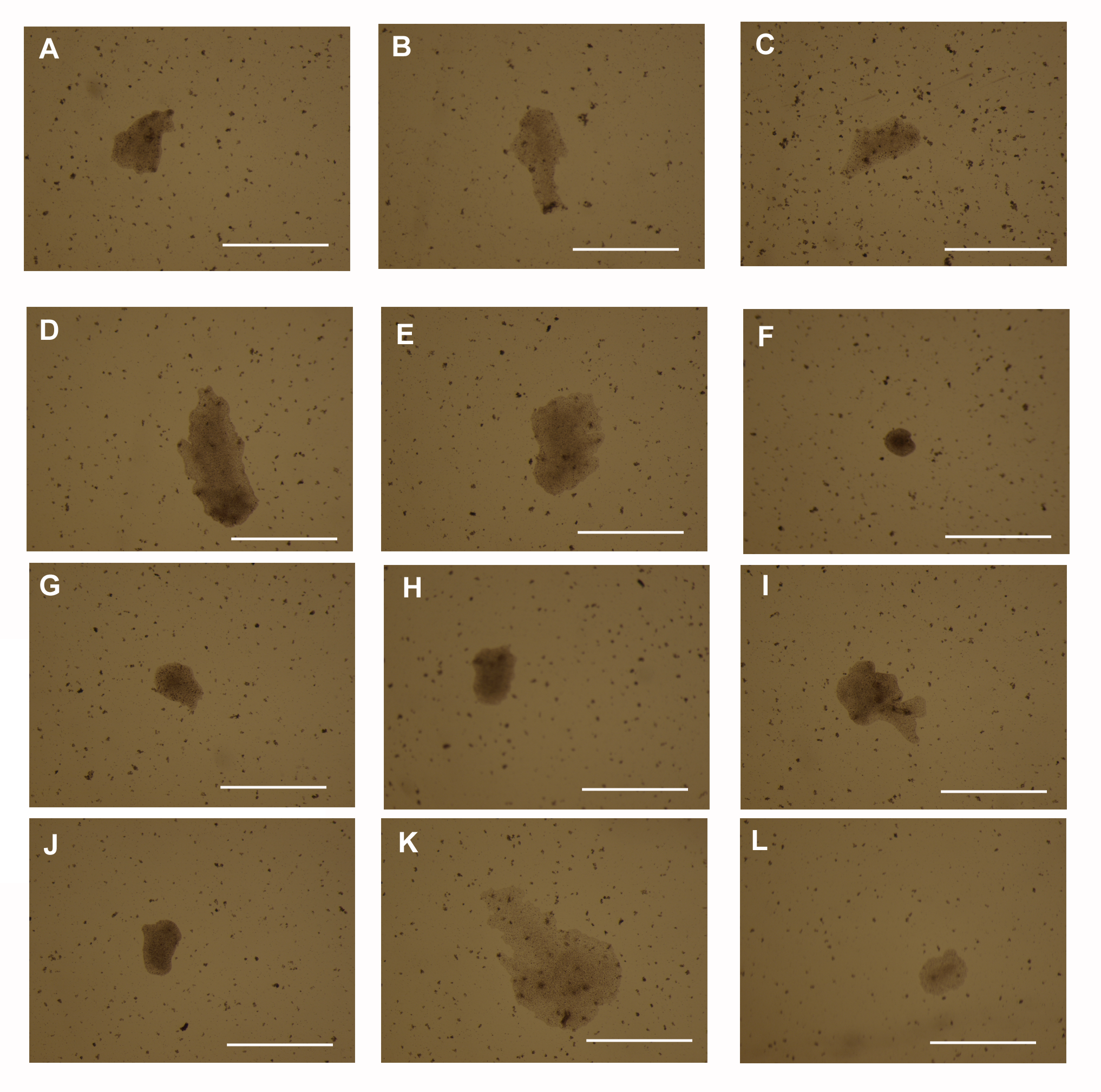

Supplement: Figure S3 — Panels A to C show three animals from the control group incubated with vehicle after 90 h. Panels D to l show animals in the culture treated by 3.3 nM 9-cis-RA for 90 h. Contrary to the culture of animals fed by Porphyridium cruentum (Fig. S1), animals fed by Chlorella sp. responded positively to the treatment by 3.3 nM 9-cis-RA, none of them developed the balloon-like phenotype and more animals survived the transfer from stationary culture to the culture fed by Chlorella sp. than the control group treated solely with the vehicle. Bars represent 1 mm. [file peerj-05-3789-s011.png]

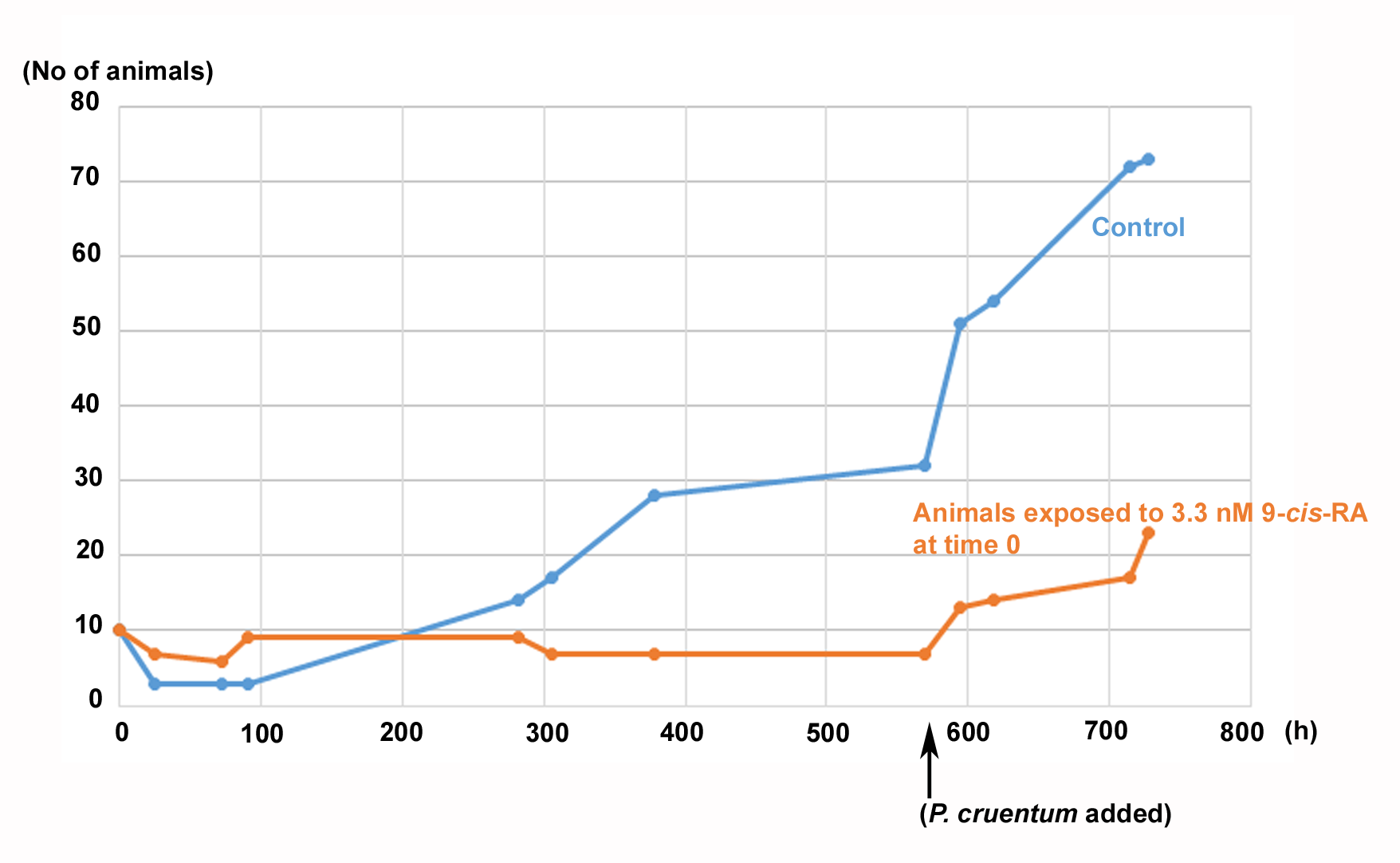

Supplement: Figure S4 — The animal cultures (started from stationary cultures fed by a naturally established milieu in a laboratory aquarium) were transferred to new Petri dishes and left for 6h to settle down. The cultures were photographed for measuring the surface and perimeter of the animals, subsequently fed with a suspension of Chlorella sp., and exposed to 3.3 nM 9-cis-RA or hormone-free vehicle; here the time was set as ‘time 0’. The cultures were incubated for 24 h in the dark, photographed again for the surface and perimeter measurements and cultivated for an additional 24 days at natural illumination in the laboratory (location: 49.98167N, 14.48725E with windows oriented WbS (azimuth 260°)). The slow growth visible in the control culture between 380 h and approximately 550 h corresponded to cloudy days and the faster growth corresponded to sunny days. After 24 days of incubation the cultures received additional feeding with Porphyridium cruentum which stimulated growth of both, the control culture and the culture exposed to 9-cis-RA (without a change of the culture medium). The experiment shows that animals arrested by the treatment with 9-cis-RA were arrested in growth but retained the potential to proliferate in response to feeding by P. cruentum. It has to be taken into account that several uncontrolled factors are likely to influence the growth of both cultures such as degradation or isomerization of 9-cis-RA in the experimental culture, or the possible growth of additional microorganisms in prolonged cultures. Natural illumination is also variable despite that external and internal lamellar window blinds were used for prevention of direct exposure of cultures to afternoon sunshine. [file peerj-05-3789-s012.png]

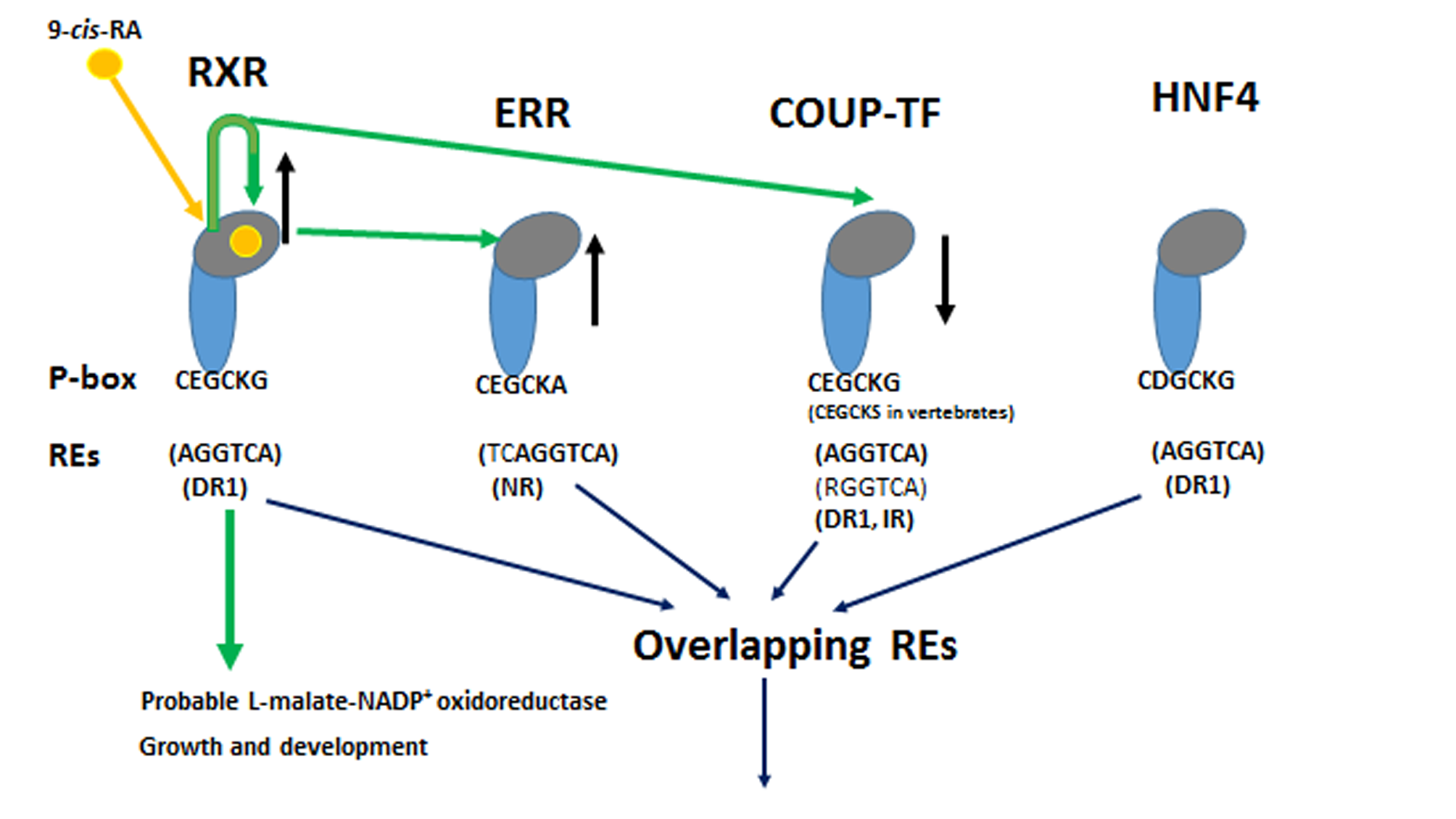

Supplement: Figure S5 — The network of T. adhaerens NRs contains four members of the NR2 subfamily. 9-cis-RA binds TaRXR which in turn up-regulates its own expression. Two additional members of the T. adhaerens NR complement respond to 9-cis-RA at the level of gene expression; ERR is up-regulated, while COUP-TF, which is often acting as a repressor of RXR dependent gene expression is down-regulated. HNF4 doesn’t seem to be regulated by 9-cis-RA at the level of its mRNA expression. All four members of T. adhaerens NR complement are likely to regulate promoters containing identical or similar response elements with the core sequence AGGTCA and thus compete or cooperate at the level of regulated promoters. Response elements stand for direct repeat 1 (DR1), non-repeats (NR) or inverted repeats (IR). [file peerj-05-3789-s013.png]
