# Supplementary material for: Trichoplax adhaerens reveals a network of nuclear receptors sensitive to 9-cis-retinoic acid at the base of metazoan evolution
Source: PeerJ. 2017 Sep 29;5:e3789. doi: 10.7717/peerj.3789 (PMC5624297; doi:10.7717/peerj.3789)
Supplement: File S7 — Assessment of the quality of GST-RXR by polyacrylamide gel electrophoresis. [file peerj-05-3789-s008.docx]

**Supplementary File S3 – Additional information concerning the analysis of the binding of 9-*cis*-RA to *Trichoplax adhaerens* RXR. Assessment of the quality of GST-RXR by polyacrylamide gel electrophoresis.**

Working assessment of the quality of bacterially expressed GST-TaRXR and thrombin cleaved TaRXR

1. Preparation of GST-RXR


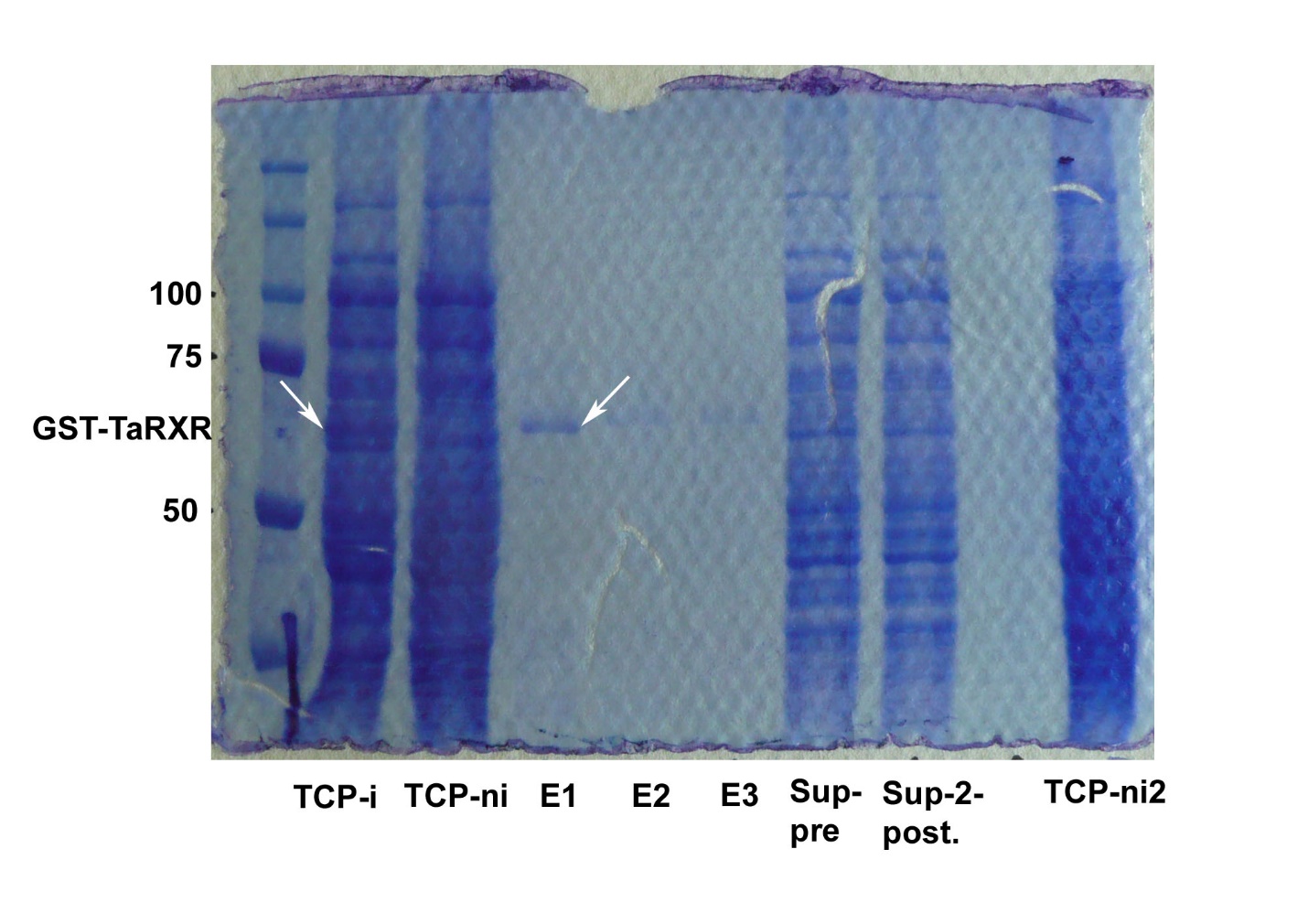


Fig. 1. Working assessment of the quality of prepared GST-RXR by polyacrylamide gel electrophoresis of total cell protein (TCP) obtained from lysed bacteria transformed with plasmid coding for GST-TaRXR after induction with IPTG (TCP-i) and noninduced (TCP-ni), protein preparations obtained after three consecutive treatments with 10mM glutathione (eluates 1 to 3 marked as E1, E2, E3), aliquots of suspension before pull-down (Sup-pre) and after pull-down (Sup-2-post.) and repeated loading of TCP from noninduced bacteria (TCP-ni2). Arrows indicate induced production of GST-TaRXR in total cell protein (TCP-i) and the first elution after the treatment with glutathione (E1).

1. Working assessment of the quality of thrombin cleaved TaRXR


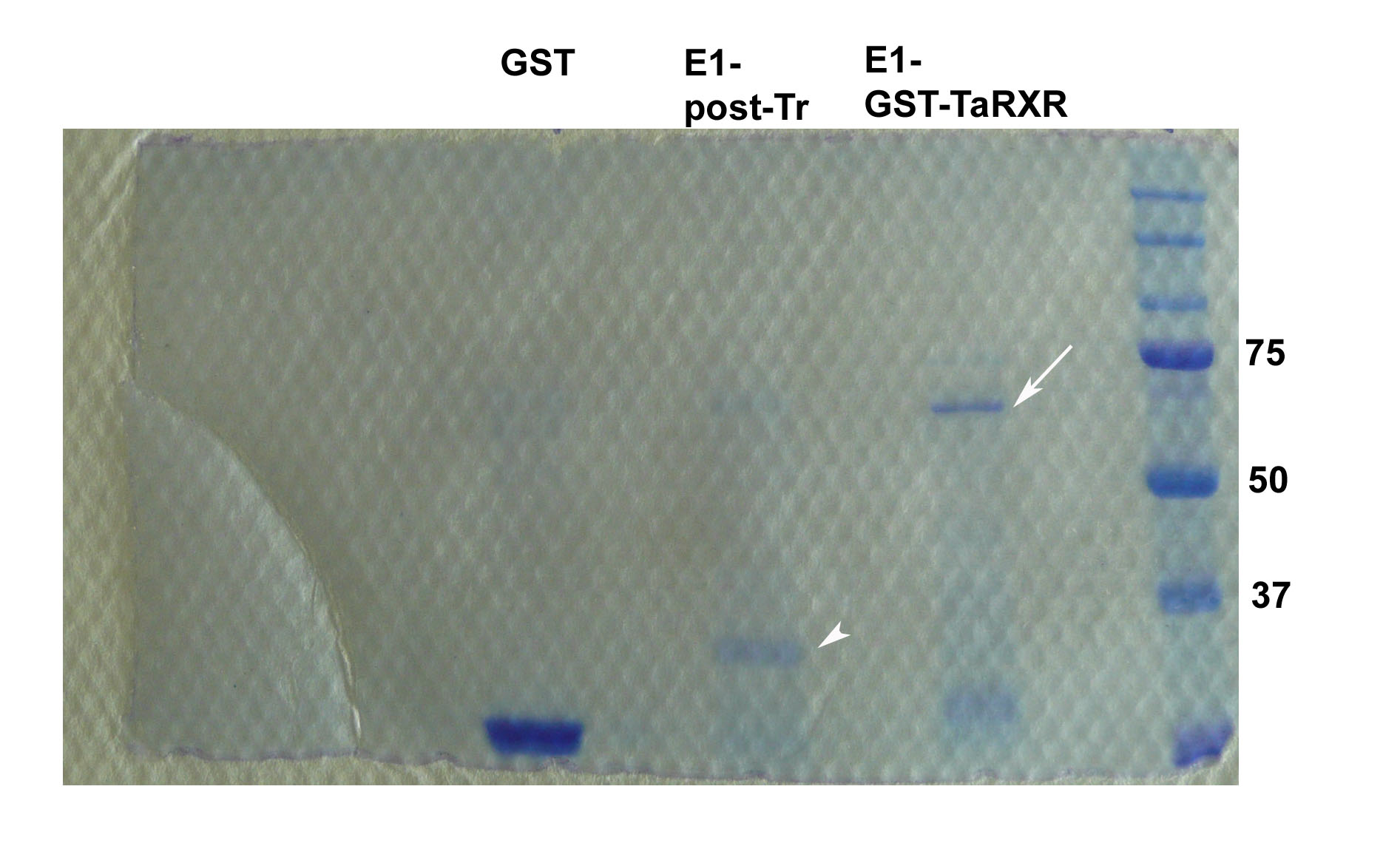


Fig. 2 Working assessment of the quality of prepared thrombin cleaved TaRXR by polyacrylamide gel electrophoresis. The GST-TaRXR bound to glutathione-agarose beads was treated by thrombin and eluted as E1-post thrombin fraction (E1-post-Tr) (arrowhead). GST prepared from the vector without inserted sequence for TaRXR and GST-TaRXR are loaded as control (E1-GST-TaRXR). TaRXR migrates as a smaller protein than expected (39 kDa).
